# Red cell specifications for blood group matching in patients with haemoglobinopathies: An updated systematic review and clinical practice guideline from the International Collaboration for Transfusion Medicine Guidelines

**DOI:** 10.1111/bjh.19837

**Published:** 2024-11-13

**Authors:** Julia Wolf, Isabelle Blais‐Normandin, Aarti Bathla, Homa Keshavarz, Stella T. Chou, Arwa Z. Al‐Riyami, Cassandra D. Josephson, Edwin Massey, Heather A. Hume, Jacob Pendergrast, Gregory Denomme, Rada M. Grubovic Rastvorceva, Sara Trompeter, Simon J. Stanworth, the International Collaboration for Transfusion Medicine Guidelines (ICTMG)

**Affiliations:** ^1^ Bristol Haematology and Oncology Centre Bristol UK; ^2^ Canadian Blood Services Vancouver British Columbia Canada; ^3^ Université Laval Québec Quebec Canada; ^4^ Canadian Blood Services Toronto Ontario Canada; ^5^ Canadian Blood Services Ottawa Ontario Canada; ^6^ Division of Hematology The Children's Hospital of Philadelphia Philadelphia Pennsylvania USA; ^7^ Department of Hematology Sultan Qaboos University Hospital, University Medical City Al Koudh Oman; ^8^ Department of Oncology and Pediatrics Johns Hopkins University School of Medicine, Johns Hopkins All Children's Hospital Baltimore Maryland USA; ^9^ Welsh Blood Service Velindre University NHS Trust Pontyclun Wales UK; ^10^ Cwm Taf Morgannwg University Health Board Pontyclun Wales UK; ^11^ Département de Pédiatrie Université de Montréal, Service d'Hématologie/Oncologie, CHU Sainte‐Justine Montréal Quebec Canada; ^12^ Department of Laboratory Medicine and Pathobiology University of Toronto, University Health Network Toronto Ontario Canada; ^13^ Grifols Laboratory Solutions San Marcos Texas USA; ^14^ Institute for Transfusion Medicine of RNM Skopje North Macedonia; ^15^ Faculty of Medical Sciences, University Goce Delcev Stip North Macedonia; ^16^ NHS Blood and Transplant London UK; ^17^ University College London London UK; ^18^ University College London NHS Foundation Trust London UK; ^19^ NHS Blood and Transplant Oxford UK; ^20^ Oxford University Hospitals NHS Trust Oxford UK; ^21^ University of Oxford Oxford UK

**Keywords:** haemoglobinopathies, red cell antigens, sickle cell disease, thalassaemia

## Abstract

Red blood cell (RBC) antigen matching beyond ABO and RhD is commonly recommended for patients with sickle cell disease (SCD) and thalassaemia. We present an updated systematic literature review to inform evidence‐based guidelines on RBC matching. The Grading of Recommendations, Assessment, Development, and Evaluation (GRADE) tool was used to develop recommendations. Six new observational studies (4 prospective, 2 retrospective) were identified. The six studies reported on 583 patients in total, including cross‐over designs, with sample sizes from 10 to 343. Studies were heterogeneous, utilising varying degrees of RBC matching and different definitions for ‘extended’ matching. All reported on alloimmunisation. One study reported on molecular matching. The reported prevalence of alloimmunisation using limited matching was 0%–50% and with extended matching was 0%–24%. Eighty‐two patients were alloimmunised before study entry. The risk of bias across studies was moderate to critical. The guideline panel recommends that ABO, RhDCcEe, and K‐compatible RBCs are selected for individuals with SCD and thalassaemia, even in the absence of alloantibodies, and that RBCs which are antigen‐negative to already existing clinically significant antibodies are chosen. There is a need for comparative research to define the benefit, impact, cost‐effectiveness, and feasibility of extended RBC matching strategies to prevent alloimmunisation.

## Graphical abstract

Red blood cell (RBC) antigen matching beyond ABO and RhD is commonly recommended for patients with sickle cell disease (SCD) and thalassaemia. We present an updated systematic literature review to inform evidence‐based guidelines on RBC matching. We identified six new observational studies (four prospective, two retrospective) which reported on 583 patients in total. The risk of bias across studies was moderate to critical. There is a need for comparative research to define the benefit, impact, cost‐effectiveness, and feasibility of extended RBC matching strategies to prevent alloimmunisation.

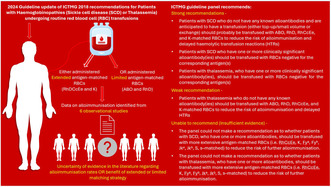

## INTRODUCTION

In sickle cell disease (SCD; including HbSS, HbSC, HbS‐beta thalassaemia, HbSD, HbSE, HbSO_Arab_) and thalassaemia, including transfusion‐dependent thalassaemia (TDT), the development of alloantibodies following red blood cell (RBC) transfusion has well‐recognised clinical consequences, including haemolytic transfusion reactions (HTRs) and delays in sourcing appropriately matched RBCs for transfusion. There is a need to understand to what degree the high prevalence of alloimmunisation in SCD and thalassaemia can be prevented by matching transfused RBCs as closely as possible to the RBC antigen phenotype of the patient.^1^, ^2^, ^3^, ^4^ The feasibility of providing more extended matched RBC units than is standard practice is dependent on many factors including the size of the donor pool, differences between donor and recipient ethnicity, and the laboratory resources available at the hospital, donor centre, and blood transfusion services. The provision of extended matched RBCs for transfusion may however be associated with delays in transfusion, which may have a clinical impact. There are also concerns that sequestering of RBC units for purposes of prophylactic matching may deplete the availability of antigen‐negative units to patients who have previously formed antibodies, placing these patients at risk of harm.

Previous evidence‐based guidelines have provided recommendations for prophylactic RBC matching beyond ABO and RhD in patients with SCD and thalassaemia aimed at reducing risks of alloimmunisation.^5^ Such practices are relevant both in patients who have never developed an alloantibody, and in those who have been previously alloimmunised, but with the added need to minimise the formation of new alloantibodies. In 2018, the International Collaboration for Transfusion Medicine Guidelines (ICTMG)^5^ made several recommendations based on a systematic review of 18 published studies (Appendix A). However, recommendations are not always consistent across guidelines, and a subsequent position paper from the British Society for Haematology (BSH)^6^ raised uncertainties about the recommendations to provide extensive phenotype‐matched RBC in patients who had acquired any RBC alloantibodies. As part of an ongoing need to review guidelines in response to new literature, this evidence‐based guideline was commissioned by ICTMG. An updated systematic review of relevant literature evidence was conducted for studies published after 2015. An international panel of experts was convened to review the new literature and to determine whether previous recommendations were still appropriate and relevant to the current clinical practice and literature or needed updating.

## METHODS

The systematic review for this guideline was conducted in accordance with 2020 PRISMA guidelines.^7^ The population of interest was people with SCD or thalassaemia, and the comparison of interest was the provision of extended matched RBC units versus restricted matching (as defined by the study). For this systematic review, we used the term extended matching to define any form of matching beyond ABO and RhD. The outcomes of interest were mortality, transfusion reactions, alloimmunisation, or mean number of RBC units transfused. Where data were unclear or missing, we attempted to contact authors. Details of the correspondence can be found in Appendix B.

### Guideline panel

The guideline panel was composed of international specialists in haematology, paediatrics and transfusion medicine with two patient representatives, and included panel members who were involved in the 2018 guideline to provide continuity of engagement. Appendix C describes the role of patient representatives in ICTMG guideline development processes. The systematic review and manuscript development was performed primarily by a smaller working group at ICTMG which was composed of three co‐chairs and a methodologist. The reporting of these guidelines was guided by AGREE REX^8^ for clinical guidelines.

### Eligibility criteria

Inclusion criteria for studies were: (1) original studies published in English with five or more patients with haemoglobinopathies (SCD or thalassaemia), (2) studies which compared different degrees of RBC matching and included any of the following outcomes: frequency of transfusion reactions or alloimmunisation, mortality, the proportion of patients transfused or the number of units transfused. Case reports, editorials, and studies published as abstracts only were excluded. Systematic or narrative reviews were manually searched for additional references.

### Information sources and search

The literature search was conducted by a library information specialist from the University Health Network (UHN), Toronto, Canada. The searches were conducted in MEDLINE, EMBASE, Cochrane Library, and CINAHL from 2016 to March 2021 and were further updated from March 2021 to August 2023. Appendix D presents the detailed search strategy. As the original guideline recommendations were published in 2018, an updated literature search also considered any change in the terminology of the key terms over the years to make sure all important and relevant references were captured.

The start date was defined by the previous guideline. References identified from bibliographic searches and additional references identified through manual searches were also included. All citations were independently assessed by two reviewers in duplicate using DistillerSR, a systematic review management software, and disagreements were resolved in consensus with the third reviewer. Similarly, data extraction was performed in duplicate for all studies, and discrepancies were resolved by mutual consensus.

### Assessing the quality of individual studies risk of bias (RoB)

Two review authors independently assessed RoB for all included studies using the risk of bias in non‐randomised studies of interventions (ROBINS‐I) tool.^9^ Discrepancies between the two reviewers were resolved by the third reviewer by mutual consensus.

### Analysis and development of recommendations

Our systematic review involved qualitative analysis of data, meta‐analysis was not conducted due to the considerable heterogeneity across studies. The Grading of Recommendations, Assessment, Development, and Evaluation (GRADE) tool^10^ was used to assess the certainty of evidence for a specific outcome. GRADE was also used to decide the strength (strong or weak/conditional) and the direction of a recommendation (support use of/does not support use of a certain intervention/matching strategy).

To grade the evidence, the working group identified the two most important outcomes by consensus. These outcomes were the development of clinically significant antibodies, and the volume/units of RBCs transfused. Evidence was downgraded if there was high RoB associated with the studies or high inconsistency within the effect estimates, or if there was indirectness, with studies not reporting directly/sufficiently on the desired outcomes, or if there was imprecision due to small sample size. Appendix E describes the definitions of High, Moderate, Low, and Very low quality of evidence as per GRADE. The strength and direction of recommendation were dependent on the certainty of evidence, the balance of benefits and harms, feasibility, acceptability of the matching strategy by the clinicians, cost and resource use considerations, patients' values and preferences, and equity.

## RESULTS

### Search results and study selection

**FIGURE 1:**
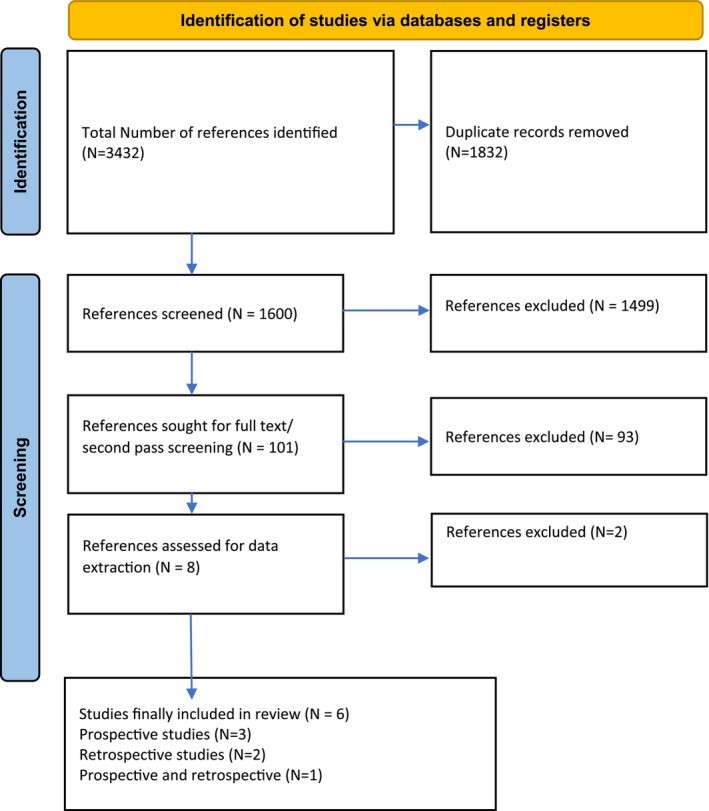
PRISMA flow chart.

A total of 3432 references were identified and screened, as presented in Figure 1. Of these, 1832 were excluded as duplicates. In total, 1600 references underwent screening review, following which 1499 were excluded. The remaining 101 references underwent full‐text review, of which 93 were excluded and eight references were considered for inclusion. Of the eight screened for full text, six references were identified for data extraction and were included in the qualitative review. All were observational studies.^11^, ^12^, ^13^, ^14^, ^15^, ^16^ The evidence for the original 2018 guideline was based on 18 references (17 clinical studies and one cost‐effectiveness study).

Of the six new studies identified, three reported on patients with a range of different thalassaemia genotypes^11^, ^13^, ^15^ (most commonly HbE/ß‐thalassaemia, then Hb H disease), one reported on patients with SCD^14^ and two studies included both populations.^12^, ^16^ Four studies were prospective observational^11^, ^12^, ^13^, ^16^ and two were retrospective observational.^14^, ^15^ Two of the six studies were from Italy,^13^, ^16^ two were from Thailand^11^, ^15^ and two were from the United States.^12^, ^14^

### Definitions of extended matching in studies

As shown in Table 1, the details of extended matching varied by study. The prospective study by Putzulu et al.^16^ compared patients transfused using extended molecular matching for ABO, RhD, RhCE, and Kell for all transfusions, as well as Fy^a/b^, Jk^a/b^, and S/s for non‐emergency transfusion, to a preceding period of non‐molecular matching, which is not well described. The prospective study by Watanaboonyongcharoen et al.^11^ described the results of extended matching of donors for ABO, RhD, RhCcEe, Mi^a^, M, S, Jk^a^, Jk^b^, Fy^a^, Fy^b^, Di^a^, and recipient genotyping for common blood group antigens.^11^ The actual matching strategy utilised was not described in detail beyond stating that patients in the intervention arm were transfused with RBCs whose phenotype was matched with the patient's genotype, whilst the control group received a standard of care matching protocol that was also not fully described. In the prospective study performed by Van Buren et al.,^12^ genotyping of both patients and donors was performed at study entry with the comparison of outcomes performed between limited (ABO, RhD, RhCcEe, K) versus extended (ABO, RhD, RhCcEe, K, Fy^a^, Fy^b^, Jk^a^, Jk^b^, S, s) matching. In the final prospective study by Belsito et al.,^13^ patients transfused following limited serological matching (ABO, RhD, RhCcEe) were compared with patients transfused following extended serological matching (ABO, RhD, RhCcEe, K, k, Fy^a^, Fy^b^, Jk^a^, Jk^b^, M, N, S, s).

**TABLE 1:** Study characteristics.

First author, year, country, centre status	Haemo‐globinopathy type	Indication for transfusion	Control vs intervention	Proportion allo‐immunised prior to study entry *N* (%)	Sample size	Mortality N (%)	Allo/auto‐immunisation rate *N* (%)	Alloantibodies developed	Follow‐up duration
						Transfusion reactions N (%)			
						Mean number of red cell units transfused N			
Putzulu, R., 2017, Italy, Single centre, Prospective and retrospective study	SCD and thalassaemia (not otherwise specified)	Periodic transfusion	Standard of care not well described versus Molecular matching for ABO, RhD, RhCE, Kell (for all) + Fy^a^/^b^, Jk^a^/^b^, Ss (for non‐emergency transfusion)	Unclear—5/10 (50%) were alloimmunised prior to starting molecular matching	*N* = 10 (Cross‐over)	NR	Limited matching: 5/10 (50%) Molecular matching: 0/10 (0%)	Anti‐S, anti‐K, anti‐E, one unable to determine	3 years
						NR			
						38 (range 5–81)			
Campbell‐Lee, S., 2018, USA, Single centre, Retrospective study	HbSC, HbSS, HbS/beta‐thal(+/0)	Ad hoc, chronic automated exchange, chronic simple transfusion	Period 1: ABO, RhD matching; subsequent matching for antigens the patient became alloimmunised against versus Period 2: ABO, RhD matching; subsequent extended prophylactic antigen matching for antigens the patient became alloimmunised against and prophylactically matched for RhCcEe, K1, S, s, Fy^a^, Fy^b^, Jk^a^, and Jk^b^	Period 1: 30/323 (9%) Period 2: 4/189 (2%) (excluded from analysis)	*N* = 147 patients receiving extended or limited matching (total *N* included in the study = 476)	NR Alloimmunised patients: Period 1: 19% Period 2: 11% Non‐alloimmunised patients: Period 1: 4% Period 2: 5%	Period 1: 110/293 (38%) following ABO, RhD matching; 48/105 (46%) following subsequent matching for antigens the patient was alloimmunised against Period 2: 48/183 (26%) following ABO, RhD matching; 10/42 (24%) following subsequent extended prophylactic matching	Anti‐E, anti‐K, anti‐C, anti‐S, anti‐Fy^a^, anti‐M, anti‐Cw, anti‐V, anti‐c, anti‐Fy^b^, anti‐e, anti‐Lu^a^, anti‐D, anti‐hrB, anti‐hrS, anti‐Kp^a^, anti‐Js^a^, anti‐U, anti‐FY3, anti‐Jk^b^, anti‐Goa, anti‐Co^b^ (Anti‐E, anti‐K and Anti‐C were the most frequently formed antibodies)	10 years
						NR			
Belsito, A., 2019, Italy, Single centre, Prospective study	Beta‐Thal major	Long‐term maintenance transfusion	Partial better matching (ABO, RhD, RhCcEe, K, k) versus Extended matching (ABO, RhD, RhCcEe, K, k, Fy^a^, Fy^b^, Jk^a^, Jk^b^, M, N, S, s)	0/18 (0%)	*N* = 18 (Cross‐over)	0%	0%	None	2 years
						0%			
						NR			
Romphruk, A.V., 2019, Thailand, Single centre, Retrospective study	Beta‐Thal/HbE, HbH, HbEA/Hb Bart, HbEA Bart/Constant‐spring, HbEA Bart/Hb Pakse	Regular transfusion	Antigen‐matched versus Partially matched (divided by antibody of interest into groups of Rh and Mia antibodies)	71/383 (19%) (excluded from analysis)	*N* = 343 patients receiving limited or extended matching Rh group *N* = 218 (143 in intervention group) Mi^a^ group *N* = 125 (24 in intervention group)	NR	Anti‐Rh 5/143 (3%) in intervention versus 4/75 (5%) in control Anti‐Mia 3/24 (13%) in intervention versus 3/101 (3%) in control	Anti‐c and anti‐E Anti‐Mi^a^	10 years
						NR			
						NR			
Watanaboonyongcharoen, P., 2020, Thailand, Single centre, Prospective study	Beta‐Thal major, Beta‐Thal/HbE, HbH/Constant spring	Long‐term maintenance transfusion	Standard of care not well described versus Transfused unit's phenotype matched to patient's genotype	20/22 (91%)	*N* = 22 (Cross‐over)	NR	*N* = 0 (0%) in intervention and control groups	None	Median follow‐up 25.5 months (10–34 months)
						NR			
						NR			
Van Buren, N.L., 2020, USA, Single centre, Prospective study	HbSC, HbSS, Beta‐Thal major, Beta‐Thal/HbE, HbH/Constant‐spring, HbAE/Constant‐spring	Long‐term maintenance transfusion but not well described	Limited (RhCcEe, K‐matched) versus Extended (RhCcEe, K, Fy^a^, Fy^b^, Jk^a^, Jk^b^, S, s matched) antigen matching	9/43 (21%)	*N* = 43	0%	Limited matching: 1/34 (3%; autoantibody; no alloantibody) Extended matching: 1/9 (11%)	Limited matching: Warm autoantibody Extended matching: Anti‐D (patient later found to have RHD*DIIIa which was not detectable by routine baseline genotyping)	3 years
						2% (N = 1/43) delayed HTR in patient receiving extended matching (personal communication)			
						NR			

Of the two retrospective studies, Campell‐Lee et al.^14^ evaluated two 5‐year time periods (period 1: 2002–2007, period 2: 2007–2012) at the same institution. In period 1, RBC units were matched for ABO and RhD for all patients, with additional matching only for any antibody that recipients formed. In period 2, there was consistent application of leucodepletion and the addition of extended prophylactic serological matching for RhD, RhCcEe, K, S, Fy, Jk antigens in patients who became alloimmunised after transfusion of ABO and RhD matched units.^14^ This study was the only study to report leucodepletion of blood. Finally, Romphruk et al.^15^ evaluated the provision of prophylactic matching based on (at a minimum) ABO, RhD, RhCcEe, and Mi^a^ antigens, on outcomes in patients with a wide range of thalassaemia genotypes.^15^

### Reported outcomes

In total, 583 patients were included in the six additionally identified studies, with sample sizes ranging from 10 to 343 patients. As one study included both SCD and thalassaemia patients, but did not indicate the number of patients receiving extended or limited RBC matching, it is not possible to ascertain how many patients of each disease group were included overall.^14^ Table 1 describes the study characteristics of all included studies in detail.

Alloimmunisation was reported as an outcome in all six studies, with 82 of the 583 patients (14.1%) alloimmunised at study entry. The reported prevalence of alloimmunisation using limited matching was 0%–50% and with extended matching was 0%–24%. Notably, in the two retrospective studies, the definition of common clinically significant alloantibodies included anti‐Mi^a^, which is uncommon outside of Asian populations.^14^, ^15^

Occurrence of transfusion reactions was reported by Belsito et al.^13^ (0% in both groups), Van Buren et al.^12^ (2%; 1/43; patient with known anti‐E receiving extended matching who developed anti‐D due to a D variant not detectable on routine genotyping), and Campbell‐Lee et al.^14^ The article by Campbell‐Lee et al.^14^ considered alloimmunised and non‐alloimmunised patients separately and found that transfusion reactions were more common in alloimmunised patients in both assessed time periods (19% vs. 4% in period 1 and 11% vs. 5% in period 2). Whilst the original study also included symptoms not related to transfusion in their calculations, true transfusion reactions (allergic, febrile non‐haemolytic transfusion reactions and HTRs) were still more common in alloimmunised patients.

Transfusion requirement after the introduction of molecular typing was reported only by Putzulu et al.,^16^ but there was no comparison to pre‐intervention requirement. Only one study (Belsito et al.)^13^ reported mortality, which was 0% in both groups. Personal communication with the author also confirmed 0% mortality in both groups in the study by Van Buren et al.^12^

Limitations of the included studies are summarised in Table 2. The study by Putzulu et al.^16^ describes its intervention in detail but it is unclear what standard of care transfusions this, presumably, heavily transfused patient cohort received prior to study entry. The study by Campbell‐Lee et al.,^14^ which compared transfusion strategies in two time periods, found no statistically significant difference in primary alloimmunisation between periods 1 and 2 but did not include a full analysis of subsequent alloimmunisation rates during both time periods. Patients in the study by Belsito et al.^13^ had received a large number of transfusions prior to study entry (mean pretransfusion period of ~14 years in a transfusion‐dependent population) and patients with pre‐existing alloantibodies were excluded from analysis. No new alloantibodies were formed during the study period. In the study by Watanaboonyongcharoen et al.,^11^ the majority of patients (20/22) were alloimmunised at study entry with 16 patients multiply alloimmunised for a total of 61 antibodies. Patients had received numerous transfusions prior to study entry with no additional antibodies formed in either group during the study period. The study by Romphruk et al.^15^ excluded patients (71/383; 19%) with pre‐existing alloantibodies in a population likely to have been multiply transfused prior to study entry.^15^ Additionally, the study antibody follow‐up included only antibodies against Rh and Mi^a^ antigens.

**TABLE 2:** Study limitations.

First author, year, country, centre status	Overview	Pre‐study period	Patient flow and outcomes	Other comments
Putzulu, R., 2017, Italy, Single centre	This study included 10 patients with thalassaemia and SCD and used extensive genotyping of a large donor population to find ‘optimally matched’ RBC units for patients	9/10 patients had received transfusions prior to the study entry period and may have been extensively transfused using strategies that are not described in the paper. 5/10 had alloantibodies prior to starting molecular matching	No new antibodies were detected after molecular matching	Standard of care is poorly described and it is not clear how heavily transfused patients were prior to study entry. However, transfusion of 5–81 RBC units over a 3‐year period in patients aged 24–76 suggests considerable transfusion burden
Campbell‐Lee, S., 2018, USA, Single centre	This study compares transfusion strategies across two time periods. Only patients forming alloantibodies were managed with extended antigen matching; others received ABO and RhD matching only The total number of patients included in this study is unclear (discrepancy between flowchart and text)	Patients with pre‐study alloantibodies were excluded. Patients are likely to have received numerous transfusions prior to study entry	In period 1 (no consistent LD; ABO and RhD matching for all patients with additional matching only for any antibody that recipients had formed) 110/293 (38%) of patients were alloimmunised, compared with 48/183 (26%) patients in period 2 (consistent LD; extended prophylactic serological matching for RhD, RhCcEe, K, S, Fy, Jk antigens in patients who were alloimmunised). Additional antibodies following limited matching were detected in 48/105 (46%) in period 1 compared with 10/42 (24%) using more extensive prophylactic matching in period 2	We noted discrepancy between the numbers in the text and those listed in Figure 1 for total N, N following exclusion and for the number of excluded patients in each arm. We attempted to contact the authors to clarify but received no response. We have therefore used the numbers from Figure 1, which appear to be more consistent After multivariable logistic regression, (primary) alloimmunisation was not statistically significantly different between period 1 and 2. Unfortunately, no such analysis was performed for subsequent antibody formation
Belsito, A., 2019, Italy, Single centre	This study included 18 patients with thalassaemia major who had a mean pre‐study transfusion period of ~14 years	Patients with pre‐study antibodies were excluded No information on pre‐study transfusion matching policies provided	No new antibodies detected during two‐year follow‐up	Study was likely biased by the selection of non‐responders (exclusion of patients with pre‐existing antibodies)—no new antibodies were to be expected, regardless of the degree of matching
Romphruk, A.V., 2019, Thailand, Single centre	This study included 383 patients with thalassaemia with a mean age of 10 years	Patients who were alloimmunised at the study entry were excluded from the analysis Of the typed patients 52% had β‐thalassaemia/HbE and were most likely multiply transfused before study entry	Study antibody follow‐up included only antibodies against Rh and Mi^a^ antigens. Overall (Rh and Mi^a^) immunisation frequency did not differ between partially matched (4.0%) and matched (4.8%). Surprisingly anti‐Mi^a^ frequency was higher in Mia matched (12.5%) compared with partially matched (3.0%) (*p* = 0.0496, Chi square test)	The study was likely biased by the selection of non‐responders (exclusion of patients with pre‐existing antibodies)
Watanaboonyongcharoen, P., 2020, Thailand, Single centre	In this study, 20/22 patients had pre‐study entry antibodies and 16 of these patients were multiply alloimmunised for a total of 61 antibodies. The median age was 24 years, and patients received transfusions every 1–4 weeks	Patients are likely to have received numerous transfusions prior to study entry	No new antibodies were detected after extended matching	The study was likely biased by the selection of non‐responders (exclusion of patients with pre‐existing antibodies)—no new antibodies were to be expected, regardless of the degree of matching Standard of care is poorly described (control arm) and results are poorly presented
Van Buren, N.L., 2020, USA, Single centre	In this study, 20 patients with SCD and 23 patients with thalassaemia, who had a mean transfusion history (RhCE and K‐matched) of 4 years (number of units not provided) before study entry were included	Pre‐study entry alloimmunisation was present in six patients with SCD and in three patients with thalassaemia, for a total of 12 alloantibodies	After the extended matched transfusion, only provided to these nine alloimmunised patients, one additional anti‐D was detected in a patient with SCD and partial D (RHD*DIIIa)	One patient receiving limited matching developed a warm autoantibody and was switched from limited matching to extended matching during the study period

Abbreviations: LD, leucodepletion; RBC, red blood cells; SCD, sickle cell disease.

### Quality of the studies

**FIGURE 2:**
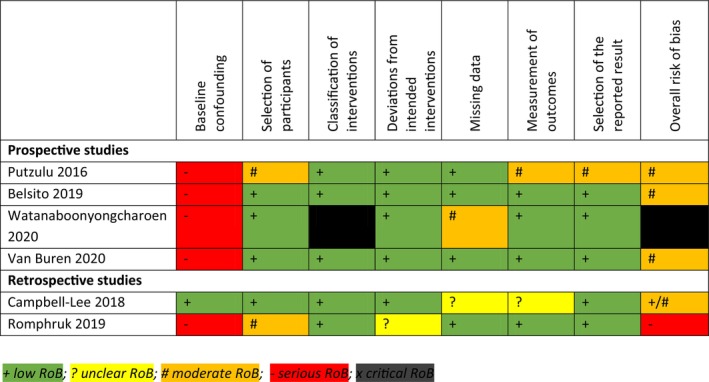
Risk of bias assessments for all studies included in the analysis (ROBINS‐I tool) (https://methods.cochrane.org/robins‐i).

The RoB assessment of the six new studies across each domain is presented in Figure 2. Critical RoB was observed in one prospective study^11^ due to bias noted in the classification of interventions; serious RoB was observed in one retrospective study^15^ due to baseline confounding. The other four studies had moderate RoB.^12^, ^13^, ^14^, ^16^ Since all studies were observational, confounding was inherently present to a degree. Furthermore, there was significant inconsistency across studies and studies did not fully describe the current standard of care.

### Patient values and preferences

For this guideline, we wanted to understand the values and preferences of patient representatives with lived experiences of haemoglobinopathies as patients, caregivers, or patient advocates. Patient representatives indicated that high value should be placed on avoiding the adverse effects of alloimmunisation associated with RBC transfusion without compromising the timely supply of blood as needed (e.g. during surgery or in emergencies). The patient representatives were aware of the value of conserving resources related to RBC transfusions and the ongoing need to ensure that the blood donor population appropriately reflected the serological needs of transfused patients, which will vary globally. Patient representatives acknowledged the importance of a diverse and appropriately sized donor pool and being able to ensure rare blood is allocated equitably, which are considerable challenges, particularly in low and middle‐resource country settings.

### GRADE summary

The GRADE evidence assessment for studies reporting the proportion of patients developing clinically significant antibodies and volume/units of RBCs transfused is presented in Table 3. As seen in the table, the certainty of evidence to recommend an extended matching strategy for both outcomes is very low. This is due to the high RoB across studies reporting on both outcomes and high inconsistency due to treatment arms not being equal in size across studies, different follow‐up times, and indirectness, as some studies did not report the current standard of care. In addition, the decreased transfusion volumes observed in patients receiving extended matching may have reflected greater reticence by physicians in transfusing this group due to the presence of their alloantibodies, or decreased availability of suitably matched units, rather than a decreased product haemolysis.

**TABLE 3:** GRADE evidence profile.

Certainty assessment							No. of patients		Effect		Certainty	Importance
No. of studies	Study design	Risk of bias	Inconsistency	Indirectness	Imprecision	Other considerations	Extended matching strategy	Limited matching strategy	Relative (95% CI)	Absolute (95% CI)		
Percentage number of patients developing clinically significant Antibodies (assessed with: Percentages)												
6	Observational studies	Serious^a^	Serious^b^	Serious^c^	Serious^d^	All plausible residual confounding would reduce the demonstrated effect	19/268 (7.1%)	60/365 (16.4%)	Not pooled	NA*	⨁◯◯◯ Very low	CRITICAL
Volume/units of RBC transfused (assessed with mean number of units transfused/per patient)												
3**	Observational studies	Serious^a^	Serious^b^	Serious^c^	Not serious	All plausible residual confounding would reduce the demonstrated effect	231	341	NA	The mean difference is reported as absolute effect Mean units for PBM = 1596.9 Mean units for EM = 865.6 Mean difference = 731.3**	⨁◯◯◯ Very low	CRITICAL

Abbreviations: CI, confidence interval; MD, mean difference.

^a^ Critical risk of bias in one study, for the proportion of patients developing clinically significant antibodies outcome, serious risk of bias in another study, and moderate risk of bias in the remaining five studies.

^b^ Considerable heterogeneity across studies, studies could not be pooled together due to this heterogeneity.

^c^ Indirectness, some studies do not report the current standard of care.

^d^ Event rate low, suggesting fragility in the estimate.

* Due to considerable heterogeneity across studies, they could not be pooled together to perform a meta‐analysis and hence there is no pooled effect estimate for this outcome.

** Putzulu et al., an observational study conducted in Italy reported the number of RBC units transfused after molecular typing but did not report the RBC transfusion requirement separately in both limited and extended matching groups, therefore, even though the data on RBC transfusion requirement is reported, these data could not be utilised to present evidence in the GRADE table as below and hence does not directly contribute towards informing the recommendation. However, this study has been described in the text for transfusion requirement outcomes.

### Recommendations

The panel reviewed and formulated recommendations in the light of the new evidence. These recommendations are summarised and compared with the 2018 ICTMG recommendations in Table 4.

**TABLE 4:** Recommendations, as per the updated evidence.

*Original recommendations*: Recommendations for RBC transfusions in patients with haemoglobinopathy	*Updated recommendations*: Red cell specifications for blood group matching in patients with haemoglobinopathies
*Recommendation 1*: Patients with SCD who do not have alloantibodies and who are anticipated to have a transfusion (simple or exchange transfusion) should probably be transfused with CcEe and K‐matched RBCs to reduce the risk of alloimmunization (low quality of evidence, weak recommendation)	*Recommendation 1* (no change): Patients with SCD who do not have any known alloantibodies and who are anticipated to have a transfusion (either top‐up/small volume or exchange) should probably be transfused with ABO, RhD, RhCcEe, and K‐matched RBCs to reduce the risk of alloimmunisation and delayed haemolytic transfusion reactions (HTRs) (low quality of evidence, strong recommendation)
*Recommendations 2*: Patients with SCD who have one or more clinically significant alloantibodies should be transfused with antigen‐negative blood to alloantibody(ies), if feasible (low quality of evidence, strong recommendation)	*Recommendation 2* (no change): Patients with SCD who have one or more clinically significant alloantibody(ies) should be transfused with RBCs negative for the corresponding antigen(s) (low quality of evidence, strong recommendation)
*Recommendation 3*: Patients with SCD who have one or more alloantibodies should probably be transfused with CcEe K Fya Fyb Jka Jkb S s matched RBCs to reduce the risk of alloimmunization, if feasible and if matching does not cause undue delays that adversely affect patient care (low quality of evidence, weak recommendation)	*Recommendation 3*: The panel could not make a recommendation as to whether patients with SCD, who have one or more alloantibodies, should be transfused with more extensive antigen‐matched RBCs (i.e. RhCcEe, K, Fy^a^, Fy^b^, Jk^a^, Jk^b^, S, s–matched) to reduce the risk of further alloimmunisation
*Recommendation 4*: Patients with thalassaemia syndromes who do not have alloantibodies and who require RBC transfusion should probably be transfused with CcEe and K‐matched RBCs to reduce the risk of alloimmunization (low quality of evidence, weak recommendation)	*Recommendation 4*: Patients with thalassaemia who do not have any known alloantibody(ies) should be transfused with ABO, RhD, RhCcEe, and K‐matched RBCs to reduce the risk of alloimmunisation and delayed HTRs (low quality of evidence, weak recommendation)
*Recommendation 5*: Patients with thalassaemia syndromes who have one or more clinically significant alloantibodies should be transfused with antigen‐negative blood to the alloantibody(ies), if feasible (low quality of evidence, strong recommendation)	*Recommendation 5*: Patients with thalassaemia, who have one or more clinically significant alloantibody(ies), should be transfused with RBCs negative for the corresponding antigen(s) (low quality of evidence, strong recommendation)
*Recommendation 6*: Patients with thalassaemia syndromes who have one or more alloantibodies should probably be transfused with CcEe K Fya Fyb Jka Jkb S s matched RBCs to reduce the risk of alloimmunization, if feasible and if matching does not cause undue delays that adversely affect patient care (low quality of evidence, weak recommendation)	*Recommendation 6*: The panel could not make a recommendation as to whether patients with thalassaemia, who have one or more alloantibodies, should be transfused with more extensive antigen‐matched RBCs (i.e. RhCcEe, K, Fy^a^, Fy^b^, Jk^a^, Jk^b^, S, s–matched) to reduce the risk of further alloimmunisation

*Recommendation 1*: Patients with SCD who do not have any known alloantibodies and who are anticipated to have a transfusion (either top‐up/small volume or exchange) should probably be transfused with ABO, RhD, RhCcEe, and K‐matched RBCs to reduce the risk of alloimmunisation and delayed haemolytic transfusion reactions (HTRs) (low quality of evidence, strong recommendation).

*Evidence summary and rationale*: Neither the updated review nor the previous one conclusively compared the outcomes of matching for ABO and RhD alone versus additional matching that has been described in publications (most commonly RhCcEe and K antigens). Although Campbell‐Lee et al.^14^ compared extended prophylactic matching for RhCcEe, Fy^a^, Fy^b^, Jk^a^, Jk^b^, K, and S to matching for antigens against which patients had alloantibodies, and demonstrated a significant reduction in alloantibody development, the apparent reduction in alloimmunisation associated with the prophylactic extended matching may have been confounded by a significantly longer follow‐up period for those who had received less extensive matching. Whilst the additional evidence available since the publication of the original guideline remains low quality, a strong recommendation is made to account for anecdotal case reports demonstrating the serious consequences of HTRs, particularly in SCD.^17^

*Recommendation 2*: Patients with SCD who have one or more clinically significant alloantibody(ies) should be transfused with RBCs negative for the corresponding antigen(s) (low quality of evidence, strong recommendation).

*Evidence summary and rationale*: No study from the updated review assessed an alternative strategy to matching for known alloantibody(ies) in SCD. However, the risk of re‐exposing patients to antigens they have previously been sensitised against has already been well‐established,^10^ with risks of HTRs, and additional antibody formation. Therefore, RBCs that are antigen‐negative to the corresponding antibody should be selected for patients who have developed a clinically significant antibody, even if this antibody is no longer currently detectable. It is unsurprising that there is limited evidence in this area as it has been a standard international practice to provide RBCs which are antigen‐negative to the corresponding antibody for many decades. Studies of transfusing RBCs with antigens to an existing alloantibody by intent would be unlikely to be ethically acceptable unless the antibody had been previously established to be clinically insignificant (e.g. via monocyte monolayer assay). Although the evidence here is weak, as in the previous guideline, a strong recommendation is made to account for case reports demonstrating the serious consequences of HTRs, particularly in SCD.^18^

*Recommendation 3*: The panel could not make a recommendation as to whether patients with SCD, who have one or more alloantibodies, should be transfused with more extensive antigen‐matched RBCs (i.e. RhCcEe, K, Fy^a^, Fy^b^, Jk^a^, Jk^b^, S, s–matched) to reduce the risk of further alloimmunisation.

*Evidence summary and rationale*: The panel concluded that there was insufficient evidence to support transfusion with RBCs prophylactically matched for Fy^a^, Fy^b^, Jk^a^, Jk^b^, S, and s antigens (in addition to ABO, RhD, RhCcEe, and K) to reduce the risk of alloimmunisation or delayed HTRs in patients with SCD. Although in the previous guideline, this was made a weak recommendation, the panel felt that the potential benefits of this approach are outweighed by its uncertain impact on resources, health economics, and provision of blood for patients who are already alloimmunised. Whilst in the newly identified studies, the overall rate of alloimmunisation was lower with extended versus more limited matching, the quality of the evidence was poor and likely subject to confounding factors. Although many centres commonly provide extended matched RBCs to SCD patients who have already been alloimmunised, this population was not well‐represented in the new studies identified in this review. In the Campbell‐Lee study,^14^ for example, previously alloimmunised patients were excluded. In the study by Van Buren et al.,^12^ only one patient was reported to be alloimmunised during the study period. Identification of factors that influence the probability of further immunisation (which would justify the provision of more extensively matched units to this population) is an area in need of further study.

*Recommendation 4*: Patients with thalassaemia who do not have any known alloantibody(ies) should be transfused with ABO, RhD, RhCcEe, and K‐matched RBCs to reduce the risk of alloimmunisation and delayed HTRs (low quality of evidence, weak recommendation).

*Evidence summary and rationale*: As with Recommendation 1 for SCD patients, no study from the updated review specifically compared limited matching (ABO and RhD) to the RhD, RhCcEe, and K‐matched approach for thalassaemia patients. The study by Belsito et al.^13^ compared limited matching (ABO, RhD, RhCcEe, and K) with extended matching in a heavily transfused and non‐alloimmunised β‐thalassaemia major population; there was no difference in allo‐ or autoimmunisation, mortality rate, and adverse transfusion reactions. The study by Van Buren et al.^12^ had a similar approach and did not demonstrate a significant difference in allo‐ or autoimmunisation. The retrospective study published by Romphruk et al.^15^ principally assessed two different matching strategies in thalassaemia patients with no previous alloimmunisation, but details were not completely described in the manuscript. The additional evidence available since the publication of the original guideline was considered insufficient to change the previous recommendation.

*Recommendation 5*: Patients with thalassaemia, who have one or more clinically significant alloantibody(ies), should be transfused with RBCs negative for the corresponding antigen(s) (low quality of evidence, strong recommendation).

*Evidence summary and rationale*: As with Recommendation 2, none of the six studies in this updated review compared an alternative matching strategy to transfusion of RBCs that are antigen‐negative to the corresponding alloantibody(ies) formed. The prior recommendation was not changed. The evidence summary and rationale for recommendation 2 for SCD patients are also relevant to patients with thalassaemia.

*Recommendation 6*: The panel could not make a recommendation as to whether patients with thalassaemia, who have one or more alloantibodies, should be transfused with more extensive antigen‐matched RBCs (i.e. RhCcEe, K, Fy^a^, Fy^b^, Jk^a^, Jk^b^, S, s–matched) to reduce the risk of further alloimmunisation.

*Evidence summary and rationale*: The panel felt there was insufficient evidence to support transfusion with RBCs prophylactically matched for RhCcEe, K, Fy^a^, Fy^b^, Jk^a^, Jk^b^, S, and s antigens to reduce the risk of alloimmunisation or delayed HTRs in patients with thalassaemia. Although in the previous guideline this was made a weak recommendation, the panel felt that, as articulated in Recommendation 3, the potential benefits of this approach are outweighed by its uncertain impact on resources, health economics, and provision of blood for patients who are already alloimmunised. The study by Watanaboonyongcharoen et al.^11^ compared two different transfusion matching strategies (ABO, RhD, RhCcEe, versus ABO, RhD, RhCcEe, Mi^a^, Fy^a^, Fy^b^, Jk^a^, Jk^b^, M, N, S, s) in a thalassaemia population in which most patients were alloimmunised prior to study entry (91%). This approach failed to show any significant difference in allo‐ or autoimmunisation, which is perhaps unsurprising given that the majority of alloantibodies are formed early in patients' transfusion history whilst patients in this study had a high number of transfusions prior to study entry. This study was also graded to have a critical RoB. Overall, there was insufficient evidence to recommend extended matching in this patient cohort.

## DISCUSSION

This updated literature review and evidence‐based clinical practice guideline continue to struggle to make recommendations for a beneficial role of extended matching of RBCs for patients with SCD and thalassaemia, a group with significant transfusion exposure and a high burden of clinically relevant alloimmunisation. This largely results from the lack of high‐quality evidence and uncertainty about whether extended‐matching reduces the risk of alloimmunisation or HTRs in this patient population. Our review has highlighted multiple research needs. Above all, well‐designed studies, with clear analysis plans for reporting alloimmunisation rates in the context of pre‐study baseline transfusion information, are required. Research priorities should include:

1. Improving our understanding of how patient factors, such as ethnicity and genetic modifiers of alloimmunisation risk (which may vary globally) and the clinical context in which transfusions are administered (e.g. episodic or chronic, elective or during acute illness) may influence the role of extended matching.
2. Identifying which patients benefit most from extended prophylactic matching and whether subgroups of patients might be better suited to different policies for extended matching.
3. Evaluating the clinical and operational impact of implementing policies for extended prophylactic matching on patients with existing alloantibodies.
4. Assessing how the advent of genotyping technologies may impact the ability to deliver extended matched RBCs and better define the need/ability to match for variants, particularly in the Rh group.^17^, ^19^
5. Ensuring new studies provide clear descriptions of the local standard of practice (in particular explaining in detail RBC matching practices), report alloimmunisation rates as prevalence and incidence to better enable comparisons between studies, and that time periods during which the antibodies are identified are clearly stated. This may be best achieved through prospective data collection and starting prior to first transfusions. In addition, new studies may want to consider collecting additional data for factors such as the age of RBC units, leukodepletion (often poorly described in studies) and haemoglobin S status of the RBC units transfused to assess how these affect transfusion outcomes in patients with haemoglobinopathies.
6. Evaluating whether the use of RBC donations from HbS carriers should be considered for all patients, even those with strict post‐transfusion HbS% targets, such as seen in the management of ischaemic stroke. Due to the difficulty in accurately measuring post‐transfusion HbS%, this is currently avoided at many centres, but the inclusion of these donations would increase the pool of donors of similar ethnicity and genotype for patients with SCD.
7. Incorporating health economic research into the adoption of more stringent or extensive prophylactic matching strategies for patients with SCD or thalassaemia. Studies need to consider the feasibility of providing alternate matching strategies in different parts of the world.
8. Establishing the selection of appropriate, consistent, and meaningful patient‐focussed outcomes for clinical research, including both benefits and risks. There continues to be a need for accurate data on adverse events related to transfusion, including transfusion reactions, timely availability of blood, and the challenges and cost implications of different matching strategies.
9. Identifying efficient ways of recruiting and retaining donors whose ethnicities correlate with patients affected by inherited RBC disorders to further minimise and manage the risks associated with alloimmunisation.

Other initiatives to reduce alloimmunisation should continue to be considered. These include patient education, the provision of alert cards, which can be presented at each hospital visit to ensure the use of appropriate antigen‐negative RBCs, and data sharing of red cell immunohematology test results between hospitals. This is especially important in the absence of centralised transfusion records, which increases the risk of delayed HTRs from antibodies which show anamnesis and are not detectable on the current antibody screen.

It should however be noted that alloantibody formation can occur despite extended RBC matching and other measures detailed above. For instance, alloantibodies may be formed in 1.2% of pregnancies, with clinically significant antibodies less commonly seen at 0.4%, in the absence of any transfusions being given.^20^ Furthermore, even extended antigen matching will not be able to match all 362 currently recognised RBC antigens.^21^ Whilst high frequency and clinically significant antigens are aimed to be included in all extended matching strategies, less frequently occurring RBC antigens still have the potential to cause alloimmunisation when discrepancies in recipient and donor RBC antigens occur.

It also should also be recognised that disparity in donor/recipient RBC antigens may vary globally. This has implications for readers and clinicians interpreting data from studies with different countries of origin. If, for example, donor and recipient populations are well matched, recipients might be more likely to get antigen‐matched units by chance, which might dilute the apparent benefit of a formal extended matching strategy.

Despite advances in genotyping as a platform to type and match RBCs for transfusion, only one of the new studies identified reported on the impact of this technology compared with phenotype‐based matching strategies, and this study was very small.^16^ Potential advantages of genotyping over serological phenotyping may include the detection of weakly‐expressed or variant antigens, detection of antigens for which no commercial antisera are available, as well as increased accuracy and decreased risk of transcription errors compared with manual testing, provided systems for electronic data transfer and reporting are available.^22^ Additionally, commercial genotyping platforms are able to detect the GATA box mutation that in almost all circumstances, prevents alloimmunisation to Fy^b^, which is commonly encountered in this patient cohort.^23^ However, the cost implications are unclear, and savings generated by avoiding the need for complex serological work‐ups^12^ need to be balanced against costs for blood centres (genotyping donors) and for hospitals (genotyping patients).

### Limitations

An important limitation of our updated guideline is the very low quality of evidence from the observational studies. We only included comparative studies in our search strategy. Large (non‐comparator) datasets presenting information on rates of alloimmunisation (e.g. antibodies per number of units transfused) over time, with clearly defined matching strategies may add additional information relevant to our questions.^18^ It should be noted that, whilst our review included both prospective and retrospective studies, our summary statistics on alloimmunisation predominantly originate from large retrospective datasets with long follow‐up. We did not capture data that have been reported to haemovigilance schemes internationally unless such data were also published in a peer reviewed journal that was subject to the searches.

### Implications for low‐middle income countries

A 2021 global survey estimated that the number of patients living with SCD was 7.74 million world‐wide (95% CI 6.5–9.2) of whom 5.7 million (95% uncertainty interval 4.8–6.6) lived in sub‐Saharan Africa (SSA),^24^ where according to one recent systematic review, transfusion for SCD is the second most common reason for transfusion overall.^25^ Approximately, 7% of transfused SCD patients in SSA may have clinically significant RBC alloantibodies.^26^ In high‐resource countries, it is often standard practice to provide patients with haemoglobinopathies with extended matched RBC units. However, this can be challenging in low/low‐middle income countries (LMIC) countries due to several factors: the genetic diversity of blood group antigens among patients and donors, limited blood supply, and scarce testing resources. This situation is exacerbated by the insufficiency of antigen testing, even for common RBC antigens, such as RhD, RhCcEe, and K, and the lack of historical records (even within single institutions) making it difficult to achieve the same standard of care in these regions. Ethnic differences between blood donors and recipients in different regions of the world may further limit the value of common generalisable recommendations. The higher prevalence of Ro (short notation of the Rh system cDe haplotype) in people of African ancestry compared with other populations may have specific implications for RhD and RhCcEe matching as potential donors from this background are more likely to be RhCcEe, as well as K, matched to patients with SCD. Resources for pretransfusion testing, including screening for clinically significant RBC alloantibodies, are often limited in many low‐resource settings, where even current WHO recommendations for pretransfusion testing may not be routinely followed.^27^

A small survey of 30 responding ISBT‐member blood providers from LMIC showed that the 2018 ICTMG haemoglobinopathy guideline recommendations for patients with SCD were often only partially followed: six centres were able to follow recommendation 1, which stated that patients without alloantibodies should probably be transfused with CcEe and K‐matched RBCs to reduce the risk of alloimmunisation; 12 centres were able to follow recommendation 2, which stated that patients with one or more clinically significant alloantibodies should be transfused with antigen‐negative blood to alloantibody(ies), if feasible; six centres were able to follow recommendation 3, which stated that patients with one or more alloantibodies should probably be transfused with CcEe, K, Fy^a^, Fy^b^, Jk^a^, Jk^b^, S, s matched RBCs to reduce the risk of alloimmunisation, if feasible and if matching does not cause undue delays that adversely affect patient care. Many respondents highlighted challenges of feasibility to implementation.^28^ Recent publications suggest that this situation is unlikely to have improved.^29^, ^30^

## CONCLUSION

The guideline panel recognised the paucity of evidence favouring the use of extended matching strategies. Well‐designed adequately resourced studies are needed to inform how to reduce alloimmunisation and hence the risks related to transfusion, especially in vulnerable patient groups who rely on transfusion as a primary treatment for their conditions. Specific matching strategies, if implemented, may need to be further adapted for different areas of the world depending on donor profiles.

## DISCLAIMER

The purpose of this document is to provide guidance on red blood cell specifications in patients with haemoglobinopathies based on the published evidence ICTMG's recommendations are not intended to replace either physicians' clinical judgement of the specific case or the physicians' personal experience.

## AUTHOR CONTRIBUTIONS

All authors had access to the included evidence base for the guideline, took responsibility for the guideline recommendations, and had authority over manuscript preparation, and the decision to submit the manuscript for publication. All listed authors meet the ICMJE authorship criteria: Substantial contributions to the conception or design of the work; or the acquisition, analysis, or interpretation of data for the work; and drafting the work or revising it critically for important intellectual content; and final approval of the version to be published; and agreement to be accountable for all aspects of the work in ensuring that questions related to the accuracy or integrity of any part of the work are appropriately investigated and resolved.

*Concept and design*: All authors. *Analysis and interpretation of the data*: JW, IBN, SS, AB, ST. *Manuscript draft*: SS, JW, IBN. *Critical revision of the manuscript for important intellectual content*: All authors. *Final approval of the manuscript*: All authors. *Administrative, technical, or logistical support*: AB, HK. *Collection and assembly of data*: HK, AB. *Provision of study material or patients*: N/A.

## FUNDING INFORMATION

This guideline was conducted on behalf of the ICTMG (https://www.ictmg.org/). ICTMG receives funding from the Canadian Blood Services which is funded by the government of Canada (Health Canada) and the provincial and territorial ministries of health. The views herein do not necessarily reflect the views of Canadian Blood Services or the federal, provincial, or territorial governments of Canada. Canadian Blood Services did not have any role in the interpretation of data or approval of the manuscript.

## CONFLICT OF INTEREST STATEMENT

All authors disclosed their potential financial, professional, or personal conflicts of interest. See Appendix F for disclosures.

## Supporting information


Data S1.
