# Supplementary material for: Red cell specifications for blood group matching in patients with haemoglobinopathies: An updated systematic review and clinical practice guideline from the International Collaboration for Transfusion Medicine Guidelines
Source: Br J Haematol. 2024 Nov 13;206(1):94–108. doi: 10.1111/bjh.19837 (PMC11739758; doi:10.1111/bjh.19837)
Supplement: Supplementary file 1 — Data S1. [file BJH-206-94-s001.docx]

**Appendices**:

Red cell specifications for blood group matching in patients with hemoglobinopathies: An updated systematic review and clinical practice guideline from the International Collaboration for Transfusion Medicine Guidelines

Table of Contents:

[Appendix A- Original guideline recommendations published in 2018 2](#_Toc174546803)

[Appendix B- Author correspondence 3](#_Toc174546804)

[Appendix C- Role of patient representative in ICTMG guideline development process 4](#_Toc174546805)

[Appendix D- Search strategy 7](#_Toc174546806)

[Appendix E- GRADE definitions 40](#_Toc174546807)

[Appendix F- Disclosures table 41](#_Toc174546808)

# Appendix A- **Original guideline recommendations published in 2018**

| ICTMG’s original recommendations for RBC transfusion in patients with hemoglobinopathies | |
| --- | --- |
| Recommendation 1 | Patients with SCD who do not have alloantibodies and who are anticipated to have a transfusion (simple or exchange transfusion) should probably be transfused with CcEe and K-matched RBCs to reduce the risk of alloimmunization (low quality of evidence, weak recommendation). |
| Recommendation 2 | Patients with SCD who have one or more clinically significant alloantibodies should be transfused with antigen negative blood to alloantibody(ies), if feasible (low quality of evidence, strong recommendation). |
| Recommendation 3 | Patients with SCD who have one or more alloantibodies should probably be transfused with CcEe K Fya Fyb Jka Jkb S s matched RBCs to reduce the risk of alloimmunization, if feasible and if matching does not cause undue delays that adversely affect patient care (low quality of evidence, weak recommendation). |
| Recommendation 4 | Patients with thalassemia syndromes who do not have alloantibodies and who require RBC transfusion should probably be transfused with CcEe and K-matched RBCs to reduce the risk of alloimmunization (low quality of evidence, weak recommendation). |
| Recommendation 5 | Patients with thalassemia syndromes who have one or more clinically significant alloantibodies should be transfused with antigen negative blood to the alloantibody(ies), if feasible (low quality of evidence, strong recommendation). |
| Recommendation 6 | Patients with thalassemia syndromes who have one or more alloantibodies should probably be transfused with CcEe K Fya Fyb Jka Jkb S s matched RBCs to reduce the risk of alloimmunization, if feasible and if matching does not cause undue delays that adversely affect patient care (low quality of evidence, weak recommendation). |
| These recommendations are in addition to standard ABO matching. | |

# Appendix **B-** Author correspondence

| Author name | Email address | Date emailed | Correspondence |
| --- | --- | --- | --- |
| A. Belsito | angela.belsito@policliniconapoli.it | 25/06/2024 | Email undelivered 25/06/2024 - no alternative email found |
| P. Watanaboon-yongcharoen | unable to find any contact information | NA | NA |
| N. Van Buren | nancy.vanburen@innovativeblood.org | 25/06/2024 | Response received on 26/06/2024 resulting in clarification of data and additional data about mortality and transfusion reactions. |
| A. Romphruk | aromphruk@gmail.com | 25/06/2024 | No response |
| S. Campbell-Lee | scampbe@uic.edu | 25/06/2024 | No response |
| R. Putzulu | rossana.putzulu@policlinicogemelli.it | 29/07/2024 | No response |

# Appendix C- Role of patient representative in ICTMG guideline development process

**Who is a patient representative?**

We use the phrase ‘patient representative’ to refer to a member of one of our Guideline Development Groups. A patient representative may be-

- An individual who has personal experience with the disease and has used healthcare services (patient) or
- An individual from the community who has indirect but extensive experience with the disease and related healthcare services (Caregiver or unpaid carer)

**What will the Guideline Development Group be doing?**

We are seeking patient involvement and engagement in one of our guideline development groups (GDG). It is vital that health care providers, professionals and researchers hear the voice of patients or caregivers.

This group will look at the available evidence and develop recommendations on Selecting red blood cells for patients with hemoglobinopathies. ICTMG has previously developed [recommendations](https://www.ictmg.org/hemoglobinopathies-1) for the same topic in 2018 and is now updating the evidence to inform the current standard of best care. If the new evidence changes practice guidance, ICTMG will update its existing recommendations accordingly.

The group is composed of professionals working in health care (e.g., hematologists, clinicians, methodologists, and other subject matter experts) and patient representatives. Patient representatives have the same status and carry out the same functions as other members.

**What knowledge and experience will I need?**

ICTMG is looking for people with an understanding of blood transfusion practices for patients with disorders of hemoglobin. These disorders are called hemoglobinopathies, and by far the most common examples are Sickle cell Disease and Thalassemia.

 As a patient representative, you will understand these diseases, either:

- Through personal experience of treatments such as blood transfusion and care provided by your healthcare system, or
- As a relative or unpaid carer of someone who has used relevant health services

You will also have:

- Good communication and team-working skills
- The ability to listen and take part in constructive debate, while being respectful of other people’s views
- Knowledge of the experiences and needs of people which gives you the ability to reflect on and champion different perspectives on this topic

**What is my role?**

- Attending one onboarding meeting (1 hour) with other patient representatives during which you will be introduced to the project, and we will discuss the importance of your perspective and how we can help you foster your engagement. This meeting will be scheduled according to your availability and other patient representatives’ availability, if required and possible. The approximate date and time for this meeting is: October 2022. The meeting will be held virtually; thus you must ensure access to internet and computer/mobile device.
- Attending one Guideline Development Group meeting (1 - 1.5 hour) with GDG members. This meeting will be scheduled according to your availability and other GDG members’ availability. The approximate date and time for this meeting is: November 2022. The meeting will be held virtually; thus you must ensure access to internet and computer/mobile device.
- Read occasional documents provided to support meetings’ discussions and comment on documents between meetings (estimated at 2 - 6 hours)
- Keep the group’s work confidential.

**What am I expected to do?**

You are expected to:

- Make sure the views, experiences and interests of patients or people who use health care services are considered by the group.
- Review topic information and the draft recommendations from a patient, service user, caregiver, or community perspective. For instance, does the information address issues important to people affected by the guidance? Does the guidance take their views or perspectives into account?
- Make sure the guidance considers people from different backgrounds.

**What’s in it for me?**

- You will be helping to make international, national, and local health care services work better for patients, people who use services, caregivers, or the public
- Being a patient representative with ICTMG shows you are an expert by experience. It also shows you can work in a team, as an equal contributor to the group alongside health care providers, researchers and other professionals
- Patient representatives may find their confidence improved, as well as developing other skills like public speaking and critical thinking

**What support will I get?**

- An ICTMG member will be available throughout your time working on this initiative, to offer help and support
- You will be offered onboarding and guidance to make sure you feel confident on the group, as well as regular chats with your named contact
- If you have any special requirements, we can discuss this with you and adjust where needed

**What happens after I apply?**

The ICTMG Secretariat passes on applications to the team running this group and you will be contacted for a short phone interview to talk about the position and what is involved, and to answer any questions you may have, before a formal invitation is offered to join the group. The date for these first calls will be in October based on the availabilities of interested individuals. They will take place on the phone and will last no more than 30 minutes.

**Will I be compensated?**

At ICTMG, we value the engagement of patients and family caregivers as we believe our work benefits from the wisdom people have gained from their lived experiences. When determining whether patient representatives may be compensated for their participation in ICTMG initiatives, our concern is to work within the principles of fair, equitable and barrier-free public engagement — and we believe this can happen either way.

The ICTMG decides on an initiative-by-initiative basis using the same decision-making process every time. The ICTMG uses “The Decision Tool – Should Money Come Into It?” developed by The Change Foundation to guide this process.

For this ICTMG initiative, the ICTM will not provide monetary compensation for patient participation. The patient representatives will be compensated through acknowledgement in the guideline publication.

**What happens if I do not join the Guideline Development Group?**

There may be other opportunities for patient participants to contribute to the work of the ICTMG, such as dissemination and implementation of recommendations. These opportunities will be presented to you should you decide not to join the Guideline Development Group or should you not be selected.

# Appendix D- Search strategy

**Search Strategies**

**Topic:**

Search update for: Compernolle V, Chou ST, Tanael S, Savage W, Howard J, Josephson CD, Odame I, Hogan C, Denomme G, Shehata N; International Collaboration for Transfusion Medicine Guidelines. Red blood cell specifications for patients with hemoglobinopathies: a systematic review and guideline. Transfusion. 2018 Jun;58(6):1555-1566.

**PRISMA Initial Results:** total number of results BEFORE duplicates removed

| **Database [Platform]** Searches run March 24, 2021.  *Results limited to September 1, 2016, to current, where possible* | **Results** |
| --- | --- |
| MEDLINE(R) ALL (Includes Epub Ahead of Print and In-Process, In-Data-Review & Other Non-Indexed Citations) [Ovid] 1946 to March 23, 2021 | 220 |
| Embase Classic+Embase 1947 to 2021 March 23 [Ovid] | 1,439 |
| Cochrane Central Register of Controlled Trials 2014 to Present [Ovid] | 10 |
| CINAHL 1981 to current [EBSCOHost] | 374 |
| **TOTAL** | **2,043** |

**PRISMA Initial Results:** total number of results AFTER duplicates removed

| **Database [Platform]** Searches run March 24, 2021.  *Results limited to September 1, 2016, forward, where possible* | **Results** |
| --- | --- |
| MEDLINE(R) ALL (Includes Epub Ahead of Print and In-Process, In-Data-Review & Other Non-Indexed Citations) [Ovid] 1946 to March 23, 2021 | 218 |
| Embase Classic+Embase 1947 to 2021 March 23 [Ovid] | 1,283 |
| Cochrane Central Register of Controlled Trials 2014 to Present [Ovid] | 2 |
| CINAHL [EBESCOHost] | 309 |
| **TOTAL** | **1,812** |

**2021 Update** - **Database Subject Headings Additions (YR Introduced) or Changes in Terms**

**MEDLINE**

Lewis X Antigen (2018) – REPLACES CD15 ANTIGENS

ADDITION: Sialyl Lewis X Antigen - 2020(2008)

Polymorphism, Genetic

ADDITION: Pharmacogenomic Variants (2018)

**Embase**

alpha thalassemia

ADDITION: alpha thalassemia-mental retardation syndrome X-linked (2019)

random amplified polymorphic DNA

ADDITION: random amplified polymorphic DNA polymerase chain reaction (2019)

pleiotropy

ADDITION: antagonistic pleiotropy (2019)

chromatin immunoprecipitation

ADDITION: chromatin immunoprecipitation polymerase chain reaction (2019)

ADDITION: chromatin immunoprecipitation sequencing (2020)

multiplex ligation dependent probe amplification

ADDITION: methylation-specific multiplex ligastion-dependent probe amplification (2019)

multiplex polymerase chain reaction

ADDITION:  multiplex real time polymerase chain reaction (2019)

ADDITION:  multiplex reverse transcription polymerase chain reaction (2019)

TUNEL assay – REPLACES nick end labeling/

random amplified polymorphic dna

ADDITION:  random amplified polymorphic DNA polymerase chain reaction (2019)

real time polymerase chain reaction

ADDITION:  allele specific real time polymerase chain reaction (2019)

ADDITION:  fluorescence quantitative polymerase chain reaction (2019)

ADDITION:  multiplex real time polymerase chain reaction (2019)

ADDITION:  quantitative methylation specific polymerase chain reaction (2019)

ADDITION:  polymerase chain reaction restriction fragment length polymorphism (2019)

restriction fragment length polymorphism

ADDITION:  real time reverse transcription polymerase chain reaction (2019)

 reverse transcription polymerase chain reaction

ADDITION:  reverse transcription polymerase chain reaction (2019)

ADDITION:  real time reverse transcription polymerase chain reaction (2019)

enzyme linked immunosorbent assay

ADDITION:  ferritin ELISA (2018)

restriction fragment length polymorphism

ADDITION:  polymerase chain reaction restriction fragment length polymorphism (2019)

**CINAHL**

Polymorphism, Genetic

ADDITION:  Polymorphism, Single Nucleotide (2020)

**MEDLINE(R) ALL**1946 to March 23, 2021
Search Strategy:

| **#** | **Searches** | **Results** |
| --- | --- | --- |
| 1 | hemoglobinopathies/ or anemia, sickle cell/ or acute chest syndrome/ or hemoglobin sc disease/ or sickle cell trait/ or hemoglobin c disease/ or thalassemia/ or alpha-thalassemia/ or beta-thalassemia/ or delta-thalassemia/ | 43932 |
| 2 | (hemoglobinopath* or (sickle adj2 (cell or cells or trait* or anemia* or anaemia*)) or thalassemi*).mp. | 54372 |
| 3 | 1 or 2 | 54531 |
| 4 | blood group antigens/ or abo blood-group system/ or duffy blood-group system/ or i blood-group system/ or kell blood-group system/ or kidd blood-group system/ or lewis blood group antigens/ or lewis x antigen/ or sialyl lewis x antigen/ or ca-19-9 antigen/ or lutheran blood-group system/ or mnss blood-group system/ or p blood-group system/ or rh-hr blood-group system/ | 45986 |
| 5 | (((blood or (red adj2 cell) or erythrocyte* or rbc or abo) adj2 group*) or "blood-group*").mp. | 63449 |
| 6 | exp Blood Transfusion/ | 87217 |
| 7 | Blood Banks/ | 7225 |
| 8 | or/4-7 | 153911 |
| 9 | genotype/ or genetic predisposition to disease/ or heterozygote/ or homozygote/ or phenotype/ or endophenotypes/ or genetic markers/ or genetic pleiotropy/ | 605590 |
| 10 | polymerase chain reaction/ or amplified fragment length polymorphism analysis/ or multiplex polymerase chain reaction/ or primed in situ labeling/ or random amplified polymorphic dna technique/ or real-time polymerase chain reaction/ or reverse transcriptase polymerase chain reaction/ | 453799 |
| 11 | microarray analysis/ or oligonucleotide array sequence analysis/ | 77754 |
| 12 | enzyme-linked immunosorbent assay/ or enzyme-linked immunospot assay/ or enzyme multiplied immunoassay technique/ | 151176 |
| 13 | polymorphism, genetic/ or genomic structural variation/ or dna copy number variations/ or pharmacogenomic variants/ or polymorphism, restriction fragment length/ or polymorphism, single nucleotide/ or polymorphism, single-stranded conformational/ | 279326 |
| 14 | (PCR or RFLP or ELISA or bloodchip or beadchip or hea or (human adj2 erythrocyt* adj2 antigen*) or haemagglutinat* or hemagglutinat* or agglutinat* or (polymerase adj2 chain adj2 react*) or (oligonucleotide adj2 array*) or genotyp* or phenotyp*).ti,ab. | 1612596 |
| 15 | hematologic tests/ or "blood grouping and crossmatching"/ | 14120 |
| 16 | Donor Selection/ | 3366 |
| 17 | or/9-16 | 2213813 |
| 18 | 3 and 8 and 17 | 947 |
| 19 | limit 18 to ed=20160901-20210323 | 197 |
| 20 | ("201609*" or "201610*" or "201611*" or "201612*" or "2017*" or "2018*" or "2019*" or "2020*" or "2021*").dt. | 5968317 |
| 21 | 18 and 20 | 197 |
| 22 | 19 or 21 | 220 |

**Embase Classic+Embase**1947 to 2021 March 23 
Search Strategy:

| **#** | **Searches** | **Results** |
| --- | --- | --- |
| 1 | hemoglobinopathy/ or hemoglobin c disease/ | 10325 |
| 2 | sickle cell/ or acute chest syndrome/ | 6169 |
| 3 | sickle cell anemia/ or hemoglobin sc disease/ or hemoglobin sd disease/ or sickle cell beta thalassemia/ or sickle cell crisis/ or sickle cell trait/ | 42441 |
| 4 | thalassemia/ or alpha thalassemia/ or beta thalassemia/ or delta thalassemia/ or thalassemia intermedia/ or thalassemia major/ or thalassemia minor/ | 37648 |
| 5 | (hemoglobinopath* or (sickle adj2 (cell or cells or trait* or anemia* or anaemia*)) or thalassemi*).mp. | 85716 |
| 6 | or/1-5 | 85954 |
| 7 | blood group antigen/ or blood group a antigen/ or blood group abh antigen/ or blood group b antigen/ or blood group e antigen/ or blood group h antigen/ or blood group i antigen/ or blood group o antigen/ or rhesus antigen/ or rhesus d antigen/ or sialyl lewis x antigen/ | 20985 |
| 8 | blood group system/ or blood group abo system/ or blood group duffy system/ or blood group i system/ or blood group kell system/ or blood group kidd system/ or blood group lewis system/ or blood group lutheran system/ or blood group mnss system/ or blood group p system/ or blood group rhesus system/ or blood group xg system/ | 27015 |
| 9 | lymphocyte antigen/ or cd15 antigen/ | 9302 |
| 10 | CA 19-9 antigen/ | 16136 |
| 11 | (((blood or (red adj2 cell) or erythrocyte* or rbc or abo) adj2 group*) or "blood-group*").mp. | 90161 |
| 12 | blood bank/ | 14974 |
| 13 | blood transfusion/ or blood autotransfusion/ or exp blood component therapy/ or exchange blood transfusion/ | 203010 |
| 14 | or/7-13 | 316616 |
| 15 | genotype/ or haplotype/ | 458032 |
| 16 | genetic predisposition/ or genetic resistance/ or genetic susceptibility/ | 174343 |
| 17 | genetic parameters/ | 3464 |
| 18 | genetic code/ or exp codon/ | 115886 |
| 19 | genetic risk/ | 63511 |
| 20 | genetic similarity/ | 5688 |
| 21 | mutation rate/ | 19955 |
| 22 | population genetic parameters/ or familial incidence/ or gene frequency/ or genetic variability/ | 405989 |
| 23 | heterozygosity/ or heterozygote/ | 130277 |
| 24 | homozygote/ or homozygosity/ | 82772 |
| 25 | phenotype/ or phenotypic variation/ | 600024 |
| 26 | genetic marker/ or cleaved amplified polymorphic sequence/ or dna marker/ or marker chromosome/ or marker gene/ or microsatellite marker/ or random amplified microsatellite/ or sequence characterized amplified region/ | 102731 |
| 27 | random amplified polymorphic dna/ or random amplified polymorphic dna polymerase chain reaction/ | 7872 |
| 28 | sequence characterized amplified region/ | 256 |
| 29 | inheritance/ | 31178 |
| 30 | pleiotropy/ or antagonistic pleiotropy/ | 14484 |
| 31 | nucleic acid analysis/ or amplified fragment length polymorphism/ or branched dna signal amplification assay/ or chromatin immunoprecipitation/ or differential display/ or dna determination/ or dna extraction/ or dna gel blot analysis/ or dna isolation/ or dna screening/ or dot hybridization/ or heteroduplex analysis/ or high resolution melting analysis/ or inverse polymerase chain reaction/ or ligase chain reaction/ or loop mediated isothermal amplification/ or multiplex ligation dependent probe amplification/ or multiplex polymerase chain reaction/ or nick end labeling/ or northern blotting/ or nucleic acid amplification/ or nucleic acid sequence based amplification/ or polymerase chain reaction/ or primed in situ labeling/ or random amplified microsatellite/ or random amplified polymorphic dna/ or real time polymerase chain reaction/ or restriction fragment length polymorphism/ or reverse transcription loop mediated isothermal amplification/ or reverse transcription polymerase chain reaction/ or ribonuclease protection assay/ or rna analysis/ or rna gel blot analysis/ or sequence characterized amplified region/ or "serial analysis of gene expression"/ or single strand conformation polymorphism/ or slot blot hybridization/ or southern blotting/ or southwestern blotting/ or telomeric repeat amplification protocol/ | 1215003 |
| 32 | chromatin immunoprecipitation/ or chromatin immunoprecipitation polymerase chain reaction/ or chromatin immunoprecipitation sequencing/ | 37398 |
| 33 | polymerase chain reaction/ or inverse polymerase chain reaction/ | 430599 |
| 34 | multiplex ligation dependent probe amplification/ or methylation-specific multiplex ligation-dependent probe amplification/ | 6870 |
| 35 | multiplex polymerase chain reaction/ or multiplex real time polymerase chain reaction/ or multiplex reverse transcription polymerase chain reaction/ | 17971 |
| 36 | tunel assay/ | 18276 |
| 37 | random amplified polymorphic dna/ or random amplified polymorphic dna polymerase chain reaction/ | 7872 |
| 38 | real time polymerase chain reaction/ or allele specific real time polymerase chain reaction/ or fluorescence quantitative polymerase chain reaction/ or multiplex real time polymerase chain reaction/ or quantitative methylation specific polymerase chain reaction/ or real time reverse transcription polymerase chain reaction/ | 299394 |
| 39 | restriction fragment length polymorphism/ or polymerase chain reaction restriction fragment length polymorphism/ | 59849 |
| 40 | reverse transcription polymerase chain reaction/ or multiplex reverse transcription polymerase chain reaction/ | 285314 |
| 41 | microarray analysis/ or microchip analysis/ | 74984 |
| 42 | hemoglobin analysis/ or hemoglobin determination/ | 28877 |
| 43 | immunoassay/ | 73066 |
| 44 | DNA microarray/ | 62184 |
| 45 | protein analysis/ or protein microarray/ | 256413 |
| 46 | enzyme linked immunosorbent assay/ or ferritin elisa/ | 388935 |
| 47 | enzyme linked immunospot assay/ or enzyme multiplied immunoassay technique/ | 13310 |
| 48 | genetic polymorphism/ | 126443 |
| 49 | dna polymorphism/ or amplified fragment length polymorphism/ | 74013 |
| 50 | restriction fragment length polymorphism/ or polymerase chain reaction restriction fragment length polymorphism/ | 59849 |
| 51 | single nucleotide polymorphism/ | 198794 |
| 52 | protein polymorphism/ | 6897 |
| 53 | copy number variation/ | 25335 |
| 54 | (PCR or RFLP or ELISA or bloodchip or beadchip or hea or (human adj2 erythrocyt* adj2 antigen*) or haemagglutinat* or hemagglutinat* or agglutinat* or (polymerase adj2 chain adj2 react*) or (oligonucleotide adj2 array*) or genotyp* or phenotyp*).ti,ab. | 2210346 |
| 55 | blood examination/ or blood analysis/ or blood group typing/ | 99337 |
| 56 | antibody screening/ or dna screening/ or donor selection/ | 12876 |
| 57 | or/15-56 | 3937650 |
| 58 | 6 and 14 and 57 | 4210 |
| 59 | limit 58 to dc=20160901-20210323 | 1439 |

**Cochrane Central Register of Controlled Trials**2014 to Present 
Search Strategy:

| **#** | **Searches** | **Results** |
| --- | --- | --- |
| 1 | hemoglobinopathies/ or anemia, sickle cell/ or acute chest syndrome/ or hemoglobin sc disease/ or sickle cell trait/ or hemoglobin c disease/ or thalassemia/ or alpha-thalassemia/ or beta-thalassemia/ or delta-thalassemia/ | 1059 |
| 2 | (hemoglobinopath* or (sickle adj2 (cell or cells or trait* or anemia* or anaemia*)) or thalassemi*).mp. | 3242 |
| 3 | hemoglobinopathy/ or hemoglobin c disease/ | 29 |
| 4 | sickle cell/ or acute chest syndrome/ | 33 |
| 5 | sickle cell anemia/ or hemoglobin sc disease/ or hemoglobin sd disease/ or sickle cell beta thalassemia/ or sickle cell crisis/ or sickle cell trait/ | 722 |
| 6 | thalassemia/ or alpha thalassemia/ or beta thalassemia/ or delta thalassemia/ or thalassemia intermedia/ or thalassemia major/ or thalassemia minor/ | 365 |
| 7 | (hemoglobinopath* or (sickle adj2 (cell or cells or trait* or anemia* or anaemia*)) or thalassemi*).mp. | 3242 |
| 8 | or/1-7 | 3245 |
| 9 | blood group antigens/ or abo blood-group system/ or duffy blood-group system/ or i blood-group system/ or kell blood-group system/ or kidd blood-group system/ or lewis blood-group system/ or antigens, cd15/ or ca-19-9 antigen/ or lutheran blood-group system/ or mnss blood-group system/ or p blood-group system/ or rh-hr blood-group system/ | 197 |
| 10 | blood group antigen/ or blood group a antigen/ or blood group abh antigen/ or blood group b antigen/ or blood group e antigen/ or blood group h antigen/ or blood group i antigen/ or blood group o antigen/ or rhesus antigen/ or rhesus d antigen/ or sialyl lewis x antigen/ | 25 |
| 11 | blood group system/ or blood group abo system/ or blood group duffy system/ or blood group i system/ or blood group kell system/ or blood group kidd system/ or blood group lewis system/ or blood group lutheran system/ or blood group mnss system/ or blood group p system/ or blood group rhesus system/ or blood group xg system/ | 0 |
| 12 | lymphocyte antigen/ or cd15 antigen/ | 0 |
| 13 | CA 19-9 antigen/ | 41 |
| 14 | (((blood or (red adj2 cell) or erythrocyte* or rbc or abo) adj2 group*) or "blood-group*").mp. | 9465 |
| 15 | exp blood transfusion/ | 3494 |
| 16 | Blood Banks/ | 39 |
| 17 | blood bank/ | 39 |
| 18 | blood transfusion/ or blood autotransfusion/ or exp blood component therapy/ or exchange blood transfusion/ | 1892 |
| 19 | or/9-18 | 12561 |
| 20 | genotype/ or genetic predisposition to disease/ or heterozygote/ or homozygote/ or phenotype/ or endophenotypes/ or genetic markers/ or genetic pleiotropy/ | 5588 |
| 21 | polymerase chain reaction/ or amplified fragment length polymorphism analysis/ or multiplex polymerase chain reaction/ or primed in situ labeling/ or random amplified polymorphic dna technique/ or real-time polymerase chain reaction/ or reverse transcriptase polymerase chain reaction/ | 2247 |
| 22 | microarray analysis/ or oligonucleotide array sequence analysis/ | 267 |
| 23 | enzyme-linked immunosorbent assay/ or enzyme-linked immunospot assay/ or enzyme multiplied immunoassay technique/ | 2554 |
| 24 | polymorphism, genetic/ or genomic structural variation/ or dna copy number variations/ or pharmacogenomic variants/ or polymorphism, restriction fragment length/ or polymorphism, single nucleotide/ or polymorphism, single-stranded conformational/ | 3293 |
| 25 | (PCR or RFLP or ELISA or bloodchip or beadchip or hea or (human adj2 erythrocyt* adj2 antigen*) or haemagglutinat* or hemagglutinat* or agglutinat* or (polymerase adj2 chain adj2 react*) or (oligonucleotide adj2 array*) or genotyp* or phenotyp*).ti,ab. | 45890 |
| 26 | hematologic tests/ or "blood grouping and crossmatching"/ | 231 |
| 27 | Donor Selection/ | 36 |
| 28 | genotype/ or haplotype/ | 3656 |
| 29 | genetic predisposition/ or genetic resistance/ or genetic susceptibility/ | 1175 |
| 30 | genetic parameters/ | 0 |
| 31 | genetic code/ or exp codon/ | 74 |
| 32 | genetic risk/ | 0 |
| 33 | genetic similarity/ | 0 |
| 34 | mutation rate/ | 9 |
| 35 | population genetic parameters/ or familial incidence/ or gene frequency/ or genetic variability/ | 487 |
| 36 | heterozygosity/ or heterozygote/ | 359 |
| 37 | homozygote/ or homozygosity/ | 250 |
| 38 | phenotype/ or phenotypic variation/ | 1130 |
| 39 | genetic marker/ or cleaved amplified polymorphic sequence/ or dna marker/ or marker chromosome/ or marker gene/ or microsatellite marker/ or random amplified microsatellite/ or sequence characterized amplified region/ | 357 |
| 40 | random amplified polymorphic dna/ or random amplified polymorphic dna polymerase chain reaction/ | 0 |
| 41 | sequence characterized amplified region/ | 0 |
| 42 | inheritance/ | 3 |
| 43 | pleiotropy/ or antagonistic pleiotropy/ | 0 |
| 44 | nucleic acid analysis/ or amplified fragment length polymorphism/ or branched dna signal amplification assay/ or chromatin immunoprecipitation/ or differential display/ or dna determination/ or dna extraction/ or dna gel blot analysis/ or dna isolation/ or dna screening/ or dot hybridization/ or heteroduplex analysis/ or high resolution melting analysis/ or inverse polymerase chain reaction/ or ligase chain reaction/ or loop mediated isothermal amplification/ or multiplex ligation dependent probe amplification/ or multiplex polymerase chain reaction/ or nick end labeling/ or northern blotting/ or nucleic acid amplification/ or nucleic acid sequence based amplification/ or polymerase chain reaction/ or primed in situ labeling/ or random amplified microsatellite/ or random amplified polymorphic dna/ or real time polymerase chain reaction/ or restriction fragment length polymorphism/ or reverse transcription loop mediated isothermal amplification/ or reverse transcription polymerase chain reaction/ or ribonuclease protection assay/ or rna analysis/ or rna gel blot analysis/ or sequence characterized amplified region/ or "serial analysis of gene expression"/ or single strand conformation polymorphism/ or slot blot hybridization/ or southern blotting/ or southwestern blotting/ or telomeric repeat amplification protocol/ | 1871 |
| 45 | chromatin immunoprecipitation/ or chromatin immunoprecipitation polymerase chain reaction/ or chromatin immunoprecipitation sequencing/ | 1 |
| 46 | polymerase chain reaction/ or inverse polymerase chain reaction/ | 1375 |
| 47 | multiplex ligation dependent probe amplification/ or methylation-specific multiplex ligation-dependent probe amplification/ | 20 |
| 48 | multiplex polymerase chain reaction/ or multiplex real time polymerase chain reaction/ or multiplex reverse transcription polymerase chain reaction/ | 20 |
| 49 | tunel assay/ | 0 |
| 50 | random amplified polymorphic dna/ or random amplified polymorphic dna polymerase chain reaction/ | 0 |
| 51 | real time polymerase chain reaction/ or allele specific real time polymerase chain reaction/ or fluorescence quantitative polymerase chain reaction/ or multiplex real time polymerase chain reaction/ or quantitative methylation specific polymerase chain reaction/ or real time reverse transcription polymerase chain reaction/ | 287 |
| 52 | restriction fragment length polymorphism/ or polymerase chain reaction restriction fragment length polymorphism/ | 176 |
| 53 | reverse transcription polymerase chain reaction/ or multiplex reverse transcription polymerase chain reaction/ | 0 |
| 54 | microarray analysis/ or microchip analysis/ | 32 |
| 55 | hemoglobin analysis/ or hemoglobin determination/ | 0 |
| 56 | immunoassay/ | 262 |
| 57 | DNA microarray/ | 235 |
| 58 | protein analysis/ or protein microarray/ | 40 |
| 59 | enzyme linked immunosorbent assay/ or ferritin elisa/ | 2491 |
| 60 | enzyme linked immunospot assay/ or enzyme multiplied immunoassay technique/ | 76 |
| 61 | genetic polymorphism/ | 1646 |
| 62 | dna polymorphism/ or amplified fragment length polymorphism/ | 2 |
| 63 | restriction fragment length polymorphism/ or polymerase chain reaction restriction fragment length polymorphism/ | 176 |
| 64 | single nucleotide polymorphism/ | 1496 |
| 65 | protein polymorphism/ | 0 |
| 66 | copy number variation/ | 23 |
| 67 | blood examination/ or blood analysis/ or blood group typing/ | 0 |
| 68 | antibody screening/ or dna screening/ or donor selection/ | 36 |
| 69 | or/20-68 | 50854 |
| 70 | 8 and 19 and 69 | 18 |
| 71 | limit 70 to yr="2015 -Current" | 10 |

**CINAHL**

| **#** | **Query** | **Results** |
| --- | --- | --- |
| S1 | (MH "Hemoglobinopathies") OR (MH "Anemia, Sickle Cell") OR (MH "Acute Chest Syndrome") OR (MH "Sickle Cell Trait") OR (MH "Thalassemia") OR (MH "alpha-Thalassemia") OR (MH "beta-Thalassemia") OR (MH "delta-Thalassemia") | 8,172 |
| S2 | (TX thalassemi*) OR (TX sickle N2 anaemia*) OR (TX sickle N2 anemia*) OR (TX sickle N2 trait*) OR (TX sickle N2 cell*) OR (TX hemoglobinopath*) | 16,775 |
| S3 | S1 OR S2 | 16,782 |
| S4 | (MH "Blood Grouping and Crossmatching") | 843 |
| S5 | (MH "Hematologic Tests") | 15,434 |
| S6 | (MH "Blood Groups") OR (MH "ABO Blood-Group System") OR (MH "Rh-Hr Blood-Group System") | 2,492 |
| S7 | (MH "HLA Antigens") | 4,284 |
| S8 | (TX blood N2 cell) OR (TX red N2 cell* N2 group*) OR (TX erythrocyte* N2 group*) OR (TX rbc N2 group*) OR (TX abo N2 group*) OR (TX "blood-group*") | 73,259 |
| S9 | (MH "Blood Component Transfusion") OR (MH "Erythrocyte Transfusion") OR (MH "Exchange Transfusion, Whole Blood") OR (MH "Cytapheresis") OR (MH "Blood Transfusion") | 17,251 |
| S10 | (MH "Blood Banks") | 2,309 |
| S11 | S4 OR S5 OR S6 OR S7 OR S8 OR S9 OR S10 | 104,745 |
| S12 | (MH "Genotype") OR (MH "Phenotype") OR (MH "Genetic Markers") OR (MH "Polymorphism, Genetic") OR (MH "Polymorphism, Single Nucleotide") | 67,628 |
| S13 | (MH "Alleles") | 9,050 |
| S14 | (TX genetic N2 predisposition) OR (TX heterozygote* OR homozygote* OR phenotype* OR endophenotypes*) | 63,966 |
| S15 | (MH "Polymerase Chain Reaction") OR (MH "Primed In Situ Labeling") OR (MH "Random Amplified Polymorphic DNA Technique") OR (MH "Reverse Transcriptase Polymerase Chain Reaction") | 47,963 |
| S16 | (MH "Blood Transfusion Reaction") | 909 |
| S17 | (MH "Microarray Analysis") OR (MH "Oligonucleotide Array Sequence Analysis") OR (MH "Protein Array Analysis") | 4,526 |
| S18 | (MH "Enzyme-Linked Immunosorbent Assay") | 22,193 |
| S19 | (TX PCR OR RFLP OR ELISA OR bloodchip OR beadchip OR hea) OR (TX human N2 erythrocyt* N2 antigen*) OR (TX haemagglutinat* OR hemagglutinat* OR agglutinat*) OR (TX polymerase N2 chain N2 react*) OR (TX oligonucleotide N2 array*) OR (TX genotyp* OR phenotyp*) | 210,501 |
| S20 | (MH "Blood Donors") OR (MH "Hematologic Tests") OR (MH "Blood Grouping and Crossmatching") | 20,416 |
| S21 | (TX donor* N2 select*) | 1,301 |
| S22 | S12 OR S13 OR S14 OR S15 OR S16 OR S17 OR S18 OR S19 OR S20 OR S21 | 262,520 |
| S23 | S3 AND S11 AND S22 | 1,388 |
| S24 | EM 20160831*- | 1,802,475 |
| S25 | S23 AND S24 | 374 |

**Ovid MEDLINE(R) ALL**1946 to March 23, 2021 **NEW STRATEGY vs APPENDIX STRATEGY** 
Search Strategy:

| **#** | **Searches** | **Results** |
| --- | --- | --- |
| 1 | hemoglobinopathies/ or anemia, sickle cell/ or acute chest syndrome/ or hemoglobin sc disease/ or sickle cell trait/ or hemoglobin c disease/ or thalassemia/ or alpha-thalassemia/ or beta-thalassemia/ or delta-thalassemia/ | 43932 |
| 2 | (hemoglobinopath* or (sickle adj2 (cell or cells or trait* or anemia* or anaemia*)) or thalassemi*).mp. | 54372 |
| 3 | 1 or 2 | 54531 |
| 4 | blood group antigens/ or abo blood-group system/ or duffy blood-group system/ or i blood-group system/ or kell blood-group system/ or kidd blood-group system/ or lewis blood group antigens/ or lewis x antigen/ or sialyl lewis x antigen/ or ca-19-9 antigen/ or lutheran blood-group system/ or mnss blood-group system/ or p blood-group system/ or rh-hr blood-group system/ | 45986 |
| 5 | (((blood or (red adj2 cell) or erythrocyte* or rbc or abo) adj2 group*) or "blood-group*").mp. | 63449 |
| 6 | exp Blood Transfusion/ | 87217 |
| 7 | Blood Banks/ | 7225 |
| 8 | or/4-7 | 153911 |
| 9 | genotype/ or genetic predisposition to disease/ or heterozygote/ or homozygote/ or phenotype/ or endophenotypes/ or genetic markers/ or genetic pleiotropy/ | 605590 |
| 10 | polymerase chain reaction/ or amplified fragment length polymorphism analysis/ or multiplex polymerase chain reaction/ or primed in situ labeling/ or random amplified polymorphic dna technique/ or real-time polymerase chain reaction/ or reverse transcriptase polymerase chain reaction/ | 453799 |
| 11 | microarray analysis/ or oligonucleotide array sequence analysis/ | 77754 |
| 12 | enzyme-linked immunosorbent assay/ or enzyme-linked immunospot assay/ or enzyme multiplied immunoassay technique/ | 151176 |
| 13 | polymorphism, genetic/ or genomic structural variation/ or dna copy number variations/ or pharmacogenomic variants/ or polymorphism, restriction fragment length/ or polymorphism, single nucleotide/ or polymorphism, single-stranded conformational/ | 279326 |
| 14 | (PCR or RFLP or ELISA or bloodchip or beadchip or hea or (human adj2 erythrocyt* adj2 antigen*) or haemagglutinat* or hemagglutinat* or agglutinat* or (polymerase adj2 chain adj2 react*) or (oligonucleotide adj2 array*) or genotyp* or phenotyp*).ti,ab. | 1612596 |
| 15 | hematologic tests/ or "blood grouping and crossmatching"/ | 14120 |
| 16 | Donor Selection/ | 3366 |
| 17 | or/9-16 | 2213813 |
| 18 | 3 and 8 and 17 | 947 |
| 19 | limit 18 to ed=20160901-20210323 | 197 |
| 20 | ("201609*" or "201610*" or "201611*" or "201612*" or "2017*" or "2018*" or "2019*" or "2020*" or "2021*").dt. | 5968317 |
| 21 | 18 and 20 | 197 |
| 22 | 19 or 21 | 220 |
| 23 | hemoglobinopathies/ or anemia, sickle cell/ or acute chest syndrome/ or hemoglobin sc disease/ or sickle cell trait/ or hemoglobin c disease/ or thalassemia/ or alpha-thalassemia/ or beta-thalassemia/ or delta-thalassemia/ | 43932 |
| 24 | (hemoglobinopath* or (sickle adj2 (cell or cells or trait* or anemia* or anaemia*)) or thalassemi*).mp. | 54372 |
| 25 | 23 or 24 | 54531 |
| 26 | blood group antigens/ or abo blood-group system/ or duffy blood-group system/ or i blood-group system/ or kell blood-group system/ or kidd blood-group system/ or lewis blood-group system/ or antigens, cd15/ or ca-19-9 antigen/ or lutheran blood-group system/ or mnss blood-group system/ or p blood-group system/ or rh-hr blood-group system/ | 45396 |
| 27 | (((blood or (red adj2 cell) or erythrocyte* or rbc or abo) adj2 group*) or "blood-group*").mp. | 63449 |
| 28 | exp Blood Transfusion/ or blood banks/ | 91626 |
| 29 | 26 or 27 or 28 | 153324 |
| 30 | genotype/ or genetic predisposition to disease/ or heterozygote/ or homozygote/ or phenotype/ or endophenotypes/ or genetic markers/ or genetic pleiotropy/ | 605590 |
| 31 | polymerase chain reaction/ or amplified fragment length polymorphism analysis/ or multiplex polymerase chain reaction/ or primed in situ labeling/ or random amplified polymorphic dna technique/ or real-time polymerase chain reaction/ or reverse transcriptase polymerase chain reaction/ | 453799 |
| 32 | microarray analysis/ or oligonucleotide array sequence analysis/ | 77754 |
| 33 | enzyme-linked immunosorbent assay/ or enzyme-linked immunospot assay/ or enzyme multiplied immunoassay technique/ | 151176 |
| 34 | polymorphism, genetic/ or genomic structural variation/ or dna copy number variations/ or polymorphism, restriction fragment length/ or polymorphism, single nucleotide/ or polymorphism, single-stranded conformational/ | 278793 |
| 35 | (PCR or RFLP or ELISA or bloodchip or beadchip or hea or (human adj2 erythrocyt* adj2 antigen*) or haemagglutinat* or hemagglutinat* or agglutinat* or (polymerase adj2 chain adj2 react*) or (oligonucleotide adj2 array*) or genotyp* or phenotyp*).ti,ab. | 1612596 |
| 36 | hematologic tests/ or "blood grouping and crossmatching"/ | 14120 |
| 37 | Donor Selection/ | 3366 |
| 38 | 30 or 31 or 32 or 33 or 34 or 35 or 36 or 37 | 2213613 |
| 39 | 25 and 29 and 38 | 947 |
| 40 | limit 39 to ed=20160901-20210323 | 197 |
| 41 | 20 and 39 | 197 |
| 42 | 40 or 41 [New Strategy vs Appendix Strategy – same results] | 220 |
| 43 | 22 or 42 [New Strategy vs Appendix Strategy – same results] | 220 |

**Embase Classic+Embase**1947 to 2021 March 22 **NEW STRATEGY vs APPENDIX STRATEGY** 
Search Strategy:

| **#** | **Searches** | **Results** |
| --- | --- | --- |
| 1 | hemoglobinopathy/ or hemoglobin c disease/ | 10325 |
| 2 | sickle cell/ or acute chest syndrome/ | 6169 |
| 3 | sickle cell anemia/ or hemoglobin sc disease/ or hemoglobin sd disease/ or sickle cell beta thalassemia/ or sickle cell crisis/ or sickle cell trait/ | 42429 |
| 4 | thalassemia/ or alpha thalassemia/ or beta thalassemia/ or delta thalassemia/ or thalassemia intermedia/ or thalassemia major/ or thalassemia minor/ | 37648 |
| 5 | (hemoglobinopath* or (sickle adj2 (cell or cells or trait* or anemia* or anaemia*)) or thalassemi*).mp. | 85703 |
| 6 | or/1-5 | 85941 |
| 7 | blood group antigen/ or blood group a antigen/ or blood group abh antigen/ or blood group b antigen/ or blood group e antigen/ or blood group h antigen/ or blood group i antigen/ or blood group o antigen/ or rhesus antigen/ or rhesus d antigen/ or sialyl lewis x antigen/ | 20985 |
| 8 | blood group system/ or blood group abo system/ or blood group duffy system/ or blood group i system/ or blood group kell system/ or blood group kidd system/ or blood group lewis system/ or blood group lutheran system/ or blood group mnss system/ or blood group p system/ or blood group rhesus system/ or blood group xg system/ | 27013 |
| 9 | lymphocyte antigen/ or cd15 antigen/ | 9301 |
| 10 | CA 19-9 antigen/ | 16136 |
| 11 | (((blood or (red adj2 cell) or erythrocyte* or rbc or abo) adj2 group*) or "blood-group*").mp. | 90152 |
| 12 | blood bank/ | 14972 |
| 13 | blood transfusion/ or blood autotransfusion/ or exp blood component therapy/ or exchange blood transfusion/ | 202992 |
| 14 | or/7-13 | 316587 |
| 15 | genotype/ or haplotype/ | 457944 |
| 16 | genetic predisposition/ or genetic resistance/ or genetic susceptibility/ | 174262 |
| 17 | genetic parameters/ | 3463 |
| 18 | genetic code/ or exp codon/ | 115881 |
| 19 | genetic risk/ | 63506 |
| 20 | genetic similarity/ | 5688 |
| 21 | mutation rate/ | 19947 |
| 22 | population genetic parameters/ or familial incidence/ or gene frequency/ or genetic variability/ | 405862 |
| 23 | heterozygosity/ or heterozygote/ | 130241 |
| 24 | homozygote/ or homozygosity/ | 82739 |
| 25 | phenotype/ or phenotypic variation/ | 599852 |
| 26 | genetic marker/ or cleaved amplified polymorphic sequence/ or dna marker/ or marker chromosome/ or marker gene/ or microsatellite marker/ or random amplified microsatellite/ or sequence characterized amplified region/ | 102657 |
| 27 | random amplified polymorphic dna/ or random amplified polymorphic dna polymerase chain reaction/ | 7872 |
| 28 | sequence characterized amplified region/ | 256 |
| 29 | inheritance/ | 31171 |
| 30 | pleiotropy/ or antagonistic pleiotropy/ | 14479 |
| 31 | nucleic acid analysis/ or amplified fragment length polymorphism/ or branched dna signal amplification assay/ or chromatin immunoprecipitation/ or differential display/ or dna determination/ or dna extraction/ or dna gel blot analysis/ or dna isolation/ or dna screening/ or dot hybridization/ or heteroduplex analysis/ or high resolution melting analysis/ or inverse polymerase chain reaction/ or ligase chain reaction/ or loop mediated isothermal amplification/ or multiplex ligation dependent probe amplification/ or multiplex polymerase chain reaction/ or nick end labeling/ or northern blotting/ or nucleic acid amplification/ or nucleic acid sequence based amplification/ or polymerase chain reaction/ or primed in situ labeling/ or random amplified microsatellite/ or random amplified polymorphic dna/ or real time polymerase chain reaction/ or restriction fragment length polymorphism/ or reverse transcription loop mediated isothermal amplification/ or reverse transcription polymerase chain reaction/ or ribonuclease protection assay/ or rna analysis/ or rna gel blot analysis/ or sequence characterized amplified region/ or "serial analysis of gene expression"/ or single strand conformation polymorphism/ or slot blot hybridization/ or southern blotting/ or southwestern blotting/ or telomeric repeat amplification protocol/ | 1214786 |
| 32 | chromatin immunoprecipitation/ or chromatin immunoprecipitation polymerase chain reaction/ or chromatin immunoprecipitation sequencing/ | 37388 |
| 33 | polymerase chain reaction/ or inverse polymerase chain reaction/ | 430500 |
| 34 | multiplex ligation dependent probe amplification/ or methylation-specific multiplex ligation-dependent probe amplification/ | 6870 |
| 35 | multiplex polymerase chain reaction/ or multiplex real time polymerase chain reaction/ or multiplex reverse transcription polymerase chain reaction/ | 17963 |
| 36 | tunel assay/ | 18265 |
| 37 | random amplified polymorphic dna/ or random amplified polymorphic dna polymerase chain reaction/ | 7872 |
| 38 | real time polymerase chain reaction/ or allele specific real time polymerase chain reaction/ or fluorescence quantitative polymerase chain reaction/ or multiplex real time polymerase chain reaction/ or quantitative methylation specific polymerase chain reaction/ or real time reverse transcription polymerase chain reaction/ | 299305 |
| 39 | restriction fragment length polymorphism/ or polymerase chain reaction restriction fragment length polymorphism/ | 59846 |
| 40 | reverse transcription polymerase chain reaction/ or multiplex reverse transcription polymerase chain reaction/ | 285307 |
| 41 | microarray analysis/ or microchip analysis/ | 74979 |
| 42 | hemoglobin analysis/ or hemoglobin determination/ | 28875 |
| 43 | immunoassay/ | 73055 |
| 44 | DNA microarray/ | 62166 |
| 45 | protein analysis/ or protein microarray/ | 256408 |
| 46 | enzyme linked immunosorbent assay/ or ferritin elisa/ | 388827 |
| 47 | enzyme linked immunospot assay/ or enzyme multiplied immunoassay technique/ | 13303 |
| 48 | genetic polymorphism/ | 126440 |
| 49 | dna polymorphism/ or amplified fragment length polymorphism/ | 74008 |
| 50 | restriction fragment length polymorphism/ or polymerase chain reaction restriction fragment length polymorphism/ | 59846 |
| 51 | single nucleotide polymorphism/ | 198734 |
| 52 | protein polymorphism/ | 6897 |
| 53 | copy number variation/ | 25328 |
| 54 | (PCR or RFLP or ELISA or bloodchip or beadchip or hea or (human adj2 erythrocyt* adj2 antigen*) or haemagglutinat* or hemagglutinat* or agglutinat* or (polymerase adj2 chain adj2 react*) or (oligonucleotide adj2 array*) or genotyp* or phenotyp*).ti,ab. | 2209772 |
| 55 | blood examination/ or blood analysis/ or blood group typing/ | 99336 |
| 56 | antibody screening/ or dna screening/ or donor selection/ | 12876 |
| 57 | or/15-56 | 3936814 |
| 58 | 6 and 14 and 57 | 4210 |
| 59 | limit 58 to dc=20160901-20210322 | 1439 |
| 60 | hemoglobinopathy/ or hemoglobin c disease/ or sickle cell anemia/ or hemoglobin sc disease/ or hemoglobin sd disease/ or sickle cell/ or sickle cell beta thalassemia/ or sickle cell crisis/ or sickle cell trait/ or thalassemia/ or alpha thalassemia/ or beta thalassemia/ or delta thalassemia/ or thalassemia intermedia/ or thalassemia major/ or thalassemia minor/ or acute chest syndrome/ | 80160 |
| 61 | (hemoglobinopath* or (sickle adj2 (cell or cells or trait* or anemia* or anaemia*)) or thalassemi*).mp. | 85703 |
| 62 | 60 or 61 | 85941 |
| 63 | blood group antigen/ or blood group a antigen/ or blood group abh antigen/ or blood group b antigen/ or blood group e antigen/ or blood group h antigen/ or blood group i antigen/ or blood group o antigen/ or rhesus antigen/ or rhesus d antigen/ or sialyl lewis x antigen/ or blood group system/ or blood group abo system/ or blood group duffy system/ or blood group i system/ or blood group kell system/ or blood group kidd system/ or blood group lewis system/ or blood group lutheran system/ or blood group mnss system/ or blood group p system/ or blood group rhesus system/ or blood group xg system/ or CD15 antigen/ or CA 19-9 antigen/ | 61983 |
| 64 | (((blood or (red adj2 cell) or erythrocyte* or rbc or abo) adj2 group*) or "blood-group*").mp. | 90152 |
| 65 | blood bank/ or blood transfusion/ or blood autotransfusion/ or exp blood component therapy/ or exchange blood transfusion/ | 211546 |
| 66 | 63 or 64 or 65 | 310794 |
| 67 | genotype/ or haplotype/ or genetic predisposition/ or genetic resistance/ or genetic susceptibility/ or genetic parameters/ or genetic code/ or exp codon/ or genetic risk/ or genetic similarity/ or mutation rate/ or population genetic parameters/ or familial incidence/ or gene frequency/ or genetic variability/ or heterozygosity/ or heterozygote/ or homozygosity/ or homozygote/ or phenotype/ or phenotypic variation/ or genetic marker/ or cleaved amplified polymorphic sequence/ or dna marker/ or marker chromosome/ or marker gene/ or microsatellite marker/ or random amplified microsatellite/ or random amplified polymorphic dna/ or sequence characterized amplified region/ or inheritance/ or pleiotropy/ | 1602911 |
| 68 | nucleic acid analysis/ or amplified fragment length polymorphism/ or branched dna signal amplification assay/ or chromatin immunoprecipitation/ or differential display/ or dna determination/ or dna extraction/ or dna gel blot analysis/ or dna isolation/ or dna screening/ or dot hybridization/ or heteroduplex analysis/ or high resolution melting analysis/ or inverse polymerase chain reaction/ or ligase chain reaction/ or loop mediated isothermal amplification/ or multiplex ligation dependent probe amplification/ or multiplex polymerase chain reaction/ or nick end labeling/ or northern blotting/ or nucleic acid amplification/ or nucleic acid sequence based amplification/ or polymerase chain reaction/ or primed in situ labeling/ or random amplified microsatellite/ or random amplified polymorphic dna/ or real time polymerase chain reaction/ or restriction fragment length polymorphism/ or reverse transcription loop mediated isothermal amplification/ or reverse transcription polymerase chain reaction/ or ribonuclease protection assay/ or rna analysis/ or rna gel blot analysis/ or sequence characterized amplified region/ or "serial analysis of gene expression"/ or single strand conformation polymorphism/ or slot blot hybridization/ or southern blotting/ or southwestern blotting/ or telomeric repeat amplification protocol/ | 1214786 |
| 69 | microarray analysis/ or microchip analysis/ or hemoglobin analysis/ or hemoglobin determination/ or immunoassay/ or dna microarray/ or protein analysis/ or protein microarray/ | 482719 |
| 70 | immunoassay/ or enzyme linked immunosorbent assay/ or enzyme linked immunospot assay/ or enzyme multiplied immunoassay technique/ | 463376 |
| 71 | genetic polymorphism/ or dna polymorphism/ or amplified fragment length polymorphism/ or restriction fragment length polymorphism/ or single nucleotide polymorphism/ or protein polymorphism/ or copy number variation/ | 435991 |
| 72 | (PCR or RFLP or ELISA or bloodchip or beadchip or hea or (human adj2 erythrocyt* adj2 antigen*) or haemagglutinat* or hemagglutinat* or agglutinat* or (polymerase adj2 chain adj2 react*) or (oligonucleotide adj2 array*) or genotyp* or phenotyp*).ti,ab. | 2209772 |
| 73 | blood examination/ or blood analysis/ or blood group typing/ | 99336 |
| 74 | antibody screening/ or dna screening/ or donor selection/ | 12876 |
| 75 | 67 or 68 or 69 or 70 or 71 or 72 or 73 or 74 | 3927561 |
| 76 | 62 and 66 and 75 | 4197 |
| 77 | limit 76 to dc=20160901-20210322 | 1430 |
| 78 | 62 not 6 | 0 |
| 79 | 66 not 14 | 0 |
| 80 | 75 not 57 | 0 |
| 81 | 77 not 59 | 0 |
| 82 | 59 not 77 [New strategy captures more references] | 9 |

**Cochrane Central Register of Controlled Trials**2014 to Present **NEW STRATEGY vs APPENDIX STRATEGY** 
Search Strategy:

| **#** | **Searches** | **Results** |
| --- | --- | --- |
| 1 | hemoglobinopathies/ or anemia, sickle cell/ or acute chest syndrome/ or hemoglobin sc disease/ or sickle cell trait/ or hemoglobin c disease/ or thalassemia/ or alpha-thalassemia/ or beta-thalassemia/ or delta-thalassemia/ | 1059 |
| 2 | (hemoglobinopath* or (sickle adj2 (cell or cells or trait* or anemia* or anaemia*)) or thalassemi*).mp. | 3242 |
| 3 | hemoglobinopathy/ or hemoglobin c disease/ | 29 |
| 4 | sickle cell/ or acute chest syndrome/ | 33 |
| 5 | sickle cell anemia/ or hemoglobin sc disease/ or hemoglobin sd disease/ or sickle cell beta thalassemia/ or sickle cell crisis/ or sickle cell trait/ | 722 |
| 6 | thalassemia/ or alpha thalassemia/ or beta thalassemia/ or delta thalassemia/ or thalassemia intermedia/ or thalassemia major/ or thalassemia minor/ | 365 |
| 7 | (hemoglobinopath* or (sickle adj2 (cell or cells or trait* or anemia* or anaemia*)) or thalassemi*).mp. | 3242 |
| 8 | or/1-7 | 3245 |
| 9 | blood group antigens/ or abo blood-group system/ or duffy blood-group system/ or i blood-group system/ or kell blood-group system/ or kidd blood-group system/ or lewis blood-group system/ or antigens, cd15/ or ca-19-9 antigen/ or lutheran blood-group system/ or mnss blood-group system/ or p blood-group system/ or rh-hr blood-group system/ | 197 |
| 10 | blood group antigen/ or blood group a antigen/ or blood group abh antigen/ or blood group b antigen/ or blood group e antigen/ or blood group h antigen/ or blood group i antigen/ or blood group o antigen/ or rhesus antigen/ or rhesus d antigen/ or sialyl lewis x antigen/ | 25 |
| 11 | blood group system/ or blood group abo system/ or blood group duffy system/ or blood group i system/ or blood group kell system/ or blood group kidd system/ or blood group lewis system/ or blood group lutheran system/ or blood group mnss system/ or blood group p system/ or blood group rhesus system/ or blood group xg system/ | 0 |
| 12 | lymphocyte antigen/ or cd15 antigen/ | 0 |
| 13 | CA 19-9 antigen/ | 41 |
| 14 | (((blood or (red adj2 cell) or erythrocyte* or rbc or abo) adj2 group*) or "blood-group*").mp. | 9465 |
| 15 | exp blood transfusion/ | 3494 |
| 16 | Blood Banks/ | 39 |
| 17 | blood bank/ | 39 |
| 18 | blood transfusion/ or blood autotransfusion/ or exp blood component therapy/ or exchange blood transfusion/ | 1892 |
| 19 | or/9-18 | 12561 |
| 20 | genotype/ or genetic predisposition to disease/ or heterozygote/ or homozygote/ or phenotype/ or endophenotypes/ or genetic markers/ or genetic pleiotropy/ | 5588 |
| 21 | polymerase chain reaction/ or amplified fragment length polymorphism analysis/ or multiplex polymerase chain reaction/ or primed in situ labeling/ or random amplified polymorphic dna technique/ or real-time polymerase chain reaction/ or reverse transcriptase polymerase chain reaction/ | 2247 |
| 22 | microarray analysis/ or oligonucleotide array sequence analysis/ | 267 |
| 23 | enzyme-linked immunosorbent assay/ or enzyme-linked immunospot assay/ or enzyme multiplied immunoassay technique/ | 2554 |
| 24 | polymorphism, genetic/ or genomic structural variation/ or dna copy number variations/ or pharmacogenomic variants/ or polymorphism, restriction fragment length/ or polymorphism, single nucleotide/ or polymorphism, single-stranded conformational/ | 3293 |
| 25 | (PCR or RFLP or ELISA or bloodchip or beadchip or hea or (human adj2 erythrocyt* adj2 antigen*) or haemagglutinat* or hemagglutinat* or agglutinat* or (polymerase adj2 chain adj2 react*) or (oligonucleotide adj2 array*) or genotyp* or phenotyp*).ti,ab. | 45890 |
| 26 | hematologic tests/ or "blood grouping and crossmatching"/ | 231 |
| 27 | Donor Selection/ | 36 |
| 28 | genotype/ or haplotype/ | 3656 |
| 29 | genetic predisposition/ or genetic resistance/ or genetic susceptibility/ | 1175 |
| 30 | genetic parameters/ | 0 |
| 31 | genetic code/ or exp codon/ | 74 |
| 32 | genetic risk/ | 0 |
| 33 | genetic similarity/ | 0 |
| 34 | mutation rate/ | 9 |
| 35 | population genetic parameters/ or familial incidence/ or gene frequency/ or genetic variability/ | 487 |
| 36 | heterozygosity/ or heterozygote/ | 359 |
| 37 | homozygote/ or homozygosity/ | 250 |
| 38 | phenotype/ or phenotypic variation/ | 1130 |
| 39 | genetic marker/ or cleaved amplified polymorphic sequence/ or dna marker/ or marker chromosome/ or marker gene/ or microsatellite marker/ or random amplified microsatellite/ or sequence characterized amplified region/ | 357 |
| 40 | random amplified polymorphic dna/ or random amplified polymorphic dna polymerase chain reaction/ | 0 |
| 41 | sequence characterized amplified region/ | 0 |
| 42 | inheritance/ | 3 |
| 43 | pleiotropy/ or antagonistic pleiotropy/ | 0 |
| 44 | nucleic acid analysis/ or amplified fragment length polymorphism/ or branched dna signal amplification assay/ or chromatin immunoprecipitation/ or differential display/ or dna determination/ or dna extraction/ or dna gel blot analysis/ or dna isolation/ or dna screening/ or dot hybridization/ or heteroduplex analysis/ or high resolution melting analysis/ or inverse polymerase chain reaction/ or ligase chain reaction/ or loop mediated isothermal amplification/ or multiplex ligation dependent probe amplification/ or multiplex polymerase chain reaction/ or nick end labeling/ or northern blotting/ or nucleic acid amplification/ or nucleic acid sequence based amplification/ or polymerase chain reaction/ or primed in situ labeling/ or random amplified microsatellite/ or random amplified polymorphic dna/ or real time polymerase chain reaction/ or restriction fragment length polymorphism/ or reverse transcription loop mediated isothermal amplification/ or reverse transcription polymerase chain reaction/ or ribonuclease protection assay/ or rna analysis/ or rna gel blot analysis/ or sequence characterized amplified region/ or "serial analysis of gene expression"/ or single strand conformation polymorphism/ or slot blot hybridization/ or southern blotting/ or southwestern blotting/ or telomeric repeat amplification protocol/ | 1871 |
| 45 | chromatin immunoprecipitation/ or chromatin immunoprecipitation polymerase chain reaction/ or chromatin immunoprecipitation sequencing/ | 1 |
| 46 | polymerase chain reaction/ or inverse polymerase chain reaction/ | 1375 |
| 47 | multiplex ligation dependent probe amplification/ or methylation-specific multiplex ligation-dependent probe amplification/ | 20 |
| 48 | multiplex polymerase chain reaction/ or multiplex real time polymerase chain reaction/ or multiplex reverse transcription polymerase chain reaction/ | 20 |
| 49 | tunel assay/ | 0 |
| 50 | random amplified polymorphic dna/ or random amplified polymorphic dna polymerase chain reaction/ | 0 |
| 51 | real time polymerase chain reaction/ or allele specific real time polymerase chain reaction/ or fluorescence quantitative polymerase chain reaction/ or multiplex real time polymerase chain reaction/ or quantitative methylation specific polymerase chain reaction/ or real time reverse transcription polymerase chain reaction/ | 287 |
| 52 | restriction fragment length polymorphism/ or polymerase chain reaction restriction fragment length polymorphism/ | 176 |
| 53 | reverse transcription polymerase chain reaction/ or multiplex reverse transcription polymerase chain reaction/ | 0 |
| 54 | microarray analysis/ or microchip analysis/ | 32 |
| 55 | hemoglobin analysis/ or hemoglobin determination/ | 0 |
| 56 | immunoassay/ | 262 |
| 57 | DNA microarray/ | 235 |
| 58 | protein analysis/ or protein microarray/ | 40 |
| 59 | enzyme linked immunosorbent assay/ or ferritin elisa/ | 2491 |
| 60 | enzyme linked immunospot assay/ or enzyme multiplied immunoassay technique/ | 76 |
| 61 | genetic polymorphism/ | 1646 |
| 62 | dna polymorphism/ or amplified fragment length polymorphism/ | 2 |
| 63 | restriction fragment length polymorphism/ or polymerase chain reaction restriction fragment length polymorphism/ | 176 |
| 64 | single nucleotide polymorphism/ | 1496 |
| 65 | protein polymorphism/ | 0 |
| 66 | copy number variation/ | 23 |
| 67 | blood examination/ or blood analysis/ or blood group typing/ | 0 |
| 68 | antibody screening/ or dna screening/ or donor selection/ | 36 |
| 69 | or/20-68 | 50854 |
| 70 | 8 and 19 and 69 | 18 |
| 71 | limit 70 to yr="2015 -Current" | 10 |
| 72 | hemoglobinopathies/ or anemia, sickle cell/ or acute chest syndrome/ or hemoglobin sc disease/ or sickle cell trait/ or hemoglobin c disease/ or thalassemia/ or alpha-thalassemia/ or beta-thalassemia/ or delta-thalassemia/ or hemoglobinopathy/ or hemoglobin c disease/ or sickle cell anemia/ or hemoglobin sc disease/ or hemoglobin sd disease/ or sickle cell/ or sickle cell beta thalassemia/ or sickle cell crisis/ or sickle cell trait/ or thalassemia/ or alpha thalassemia/ or beta thalassemia/ or delta thalassemia/ or thalassemia intermedia/ or thalassemia major/ or thalassemia minor/ or acute chest syndrome/ | 1059 |
| 73 | (hemoglobinopath* or (sickle adj2 (cell or cells or trait* or anemia* or anaemia*)) or thalassemi*).mp. | 3242 |
| 74 | 72 or 73 | 3245 |
| 75 | blood group antigens/ or abo blood-group system/ or duffy blood-group system/ or i blood-group system/ or kell blood-group system/ or kidd blood-group system/ or lewis blood-group system/ or antigens, cd15/ or ca-19-9 antigen/ or lutheran blood-group system/ or mnss blood-group system/ or p blood-group system/ or rh-hr blood-group system/ or blood group antigen/ or blood group a antigen/ or blood group abh antigen/ or blood group b antigen/ or blood group e antigen/ or blood group h antigen/ or blood group i antigen/ or blood group o antigen/ or rhesus antigen/ or rhesus d antigen/ or sialyl lewis x antigen/ or blood group system/ or blood group abo system/ or blood group duffy system/ or blood group i system/ or blood group kell system/ or blood group kidd system/ or blood group lewis system/ or blood group lutheran system/ or blood group mnss system/ or blood group p system/ or blood group rhesus system/ or blood group xg system/ or CD15 antigen/ or CA 19-9 antigen/ | 200 |
| 76 | (((blood or (red adj2 cell) or erythrocyte* or rbc or abo) adj2 group*) or "blood-group*").mp. | 9465 |
| 77 | exp Blood Transfusion/ or blood banks/ or blood bank/ or blood transfusion/ or blood autotransfusion/ or exp blood component therapy/ or exchange blood transfusion/ | 3517 |
| 78 | 75 or 76 or 77 | 12561 |
| 79 | genotype/ or genetic predisposition to disease/ or heterozygote/ or homozygote/ or phenotype/ or endophenotypes/ or genetic markers/ or genetic pleiotropy/ or haplotype/ or genetic predisposition/ or genetic resistance/ or genetic susceptibility/ or genetic parameters/ or genetic code/ or exp codon/ or genetic risk/ or genetic similarity/ or mutation rate/ or population genetic parameters/ or familial incidence/ or gene frequency/ or genetic variability/ or heterozygosity/ or heterozygote/ or homozygosity/ or homozygote/ or phenotype/ or phenotypic variation/ or genetic marker/ or cleaved amplified polymorphic sequence/ or dna marker/ or marker chromosome/ or marker gene/ or microsatellite marker/ or random amplified microsatellite/ or random amplified polymorphic dna/ or sequence characterized amplified region/ or inheritance/ or pleiotropy/ | 5898 |
| 80 | polymerase chain reaction/ or amplified fragment length polymorphism analysis/ or multiplex polymerase chain reaction/ or primed in situ labeling/ or random amplified polymorphic dna technique/ or real-time polymerase chain reaction/ or reverse transcriptase polymerase chain reaction/ or nucleic acid analysis/ or amplified fragment length polymorphism/ or branched dna signal amplification assay/ or chromatin immunoprecipitation/ or cleaved amplified polymorphic sequence/ or differential display/ or dna determination/ or dna extraction/ or dna gel blot analysis/ or dna isolation/ or dna screening/ or dot hybridization/ or heteroduplex analysis/ or high resolution melting analysis/ or inverse polymerase chain reaction/ or ligase chain reaction/ or loop mediated isothermal amplification/ or multiplex ligation dependent probe amplification/ or multiplex polymerase chain reaction/ or nick end labeling/ or northern blotting/ or nucleic acid amplification/ or nucleic acid sequence based amplification/ or polymerase chain reaction/ or primed in situ labeling/ or random amplified microsatellite/ or random amplified polymorphic dna/ or real time polymerase chain reaction/ or restriction fragment length polymorphism/ or reverse transcription loop mediated isothermal amplification/ or reverse transcription polymerase chain reaction/ or ribonuclease protection assay/ or rna analysis/ or rna gel blot analysis/ or sequence characterized amplified region/ or "serial analysis of gene expression"/ or single strand conformation polymorphism/ or slot blot hybridization/ or southern blotting/ or southwestern blotting/ or telomeric repeat amplification protocol/ | 2420 |
| 81 | microarray analysis/ or oligonucleotide array sequence analysis/ or microarray analysis/ or microchip analysis/ or hemoglobin analysis/ or hemoglobin determination/ or immunoassay/ or dna microarray/ or protein analysis/ or protein microarray/ | 567 |
| 82 | enzyme-linked immunosorbent assay/ or enzyme-linked immunospot assay/ or enzyme multiplied immunoassay technique/ or immunoassay/ or enzyme linked immunosorbent assay/ or enzyme linked immunospot assay/ or enzyme multiplied immunoassay technique/ | 2805 |
| 83 | polymorphism, genetic/ or genomic structural variation/ or dna copy number variations/ or polymorphism, restriction fragment length/ or polymorphism, single nucleotide/ or polymorphism, single-stranded conformational/ or genetic polymorphism/ or dna polymorphism/ or amplified fragment length polymorphism/ or restriction fragment length polymorphism/ or single nucleotide polymorphism/ or protein polymorphism/ or copy number variation/ | 3253 |
| 84 | (PCR or RFLP or ELISA or bloodchip or beadchip or hea or (human adj2 erythrocyt* adj2 antigen*) or haemagglutinat* or hemagglutinat* or agglutinat* or (polymerase adj2 chain adj2 react*) or (oligonucleotide adj2 array*) or genotyp* or phenotyp*).ti,ab. | 45890 |
| 85 | hematologic tests/ or "blood grouping and crossmatching"/ or blood examination/ or blood analysis/ or blood group typing/ | 231 |
| 86 | Donor Selection/ or antibody screening/ or dna screening/ or donor selection/ | 36 |
| 87 | 79 or 80 or 81 or 82 or 83 or 84 or 85 or 86 | 50846 |
| 88 | 74 and 78 and 87 | 18 |
| 89 | limit 88 to yr="2015 -Current" | 10 |
| 90 | 71 or 89 [New Strategy vs Appendix Strategy – same results] | 10 |

**CINAHL NEW STRATEGY vs APPENDIX STRATEGY**

| **#** | **Query** | **Limiters/Expanders** | **Results** |
| --- | --- | --- | --- |
| S49 | S25 OR S42 | Expanders - Apply equivalent subjects  Search modes - Boolean/Phrase | 433 |
| S48 | S40 NOT S22 | Expanders - Apply equivalent subjects  Search modes - Boolean/Phrase | 0 |
| S47 | S31 NOT S11 | Expanders - Apply equivalent subjects  Search modes - Boolean/Phrase | 0 |
| S46 | S28 NOT S3 | Expanders - Apply equivalent subjects  Search modes - Boolean/Phrase | 0 |
| S45 | S22 NOT S40 | Expanders - Apply equivalent subjects  Search modes - Boolean/Phrase | 59 |
| S44 | S11 NOT S31 | Expanders - Apply equivalent subjects  Search modes - Boolean/Phrase | 0 |
| S43 | S3 NOT S28 | Expanders - Apply equivalent subjects  Search modes - Boolean/Phrase | 0 |
| S42 | S24 AND S41 | Expanders - Apply equivalent subjects  Search modes - Boolean/Phrase | 433 |
| S41 | S28 AND S31 AND S40 | Expanders - Apply equivalent subjects  Search modes - Boolean/Phrase | 1,388 |
| S40 | S32 OR S33 OR S34 OR S35 OR S36 OR S37 OR S38 OR S39 | Expanders - Apply equivalent subjects  Search modes - Boolean/Phrase | 262,461 |
| S39 | (MH "Blood Donors") OR (TX donor* N2 select*) | Expanders - Apply equivalent subjects  Search modes - Boolean/Phrase | 5,434 |
| S38 | (MH "Hematologic Tests") OR (MH "Blood Grouping and Crossmatching") | Expanders - Apply equivalent subjects  Search modes - Boolean/Phrase | 16,252 |
| S37 | (TX PCR OR RFLP OR ELISA OR bloodchip OR beadchip OR hea) OR (TX human N2 erythrocyt* N2 antigen*) OR (TX haemagglutinat* OR hemagglutinat* OR agglutinat*) OR (TX polymerase N2 chain N2 react*) OR (TX oligonucleotide N2 array*) OR (TX genotyp* OR phenotyp*) | Expanders - Apply equivalent subjects  Search modes - Boolean/Phrase | 210,501 |
| S36 | (MH "Polymorphism, Genetic") | Expanders - Apply equivalent subjects  Search modes - Boolean/Phrase | 29,431 |
| S35 | (MH "Enzyme-Linked Immunosorbent Assay") | Expanders - Apply equivalent subjects  Search modes - Boolean/Phrase | 22,193 |
| S34 | (MH "Microarray Analysis") OR (MH "Oligonucleotide Array Sequence Analysis") OR (MH "Protein Array Analysis") | Expanders - Apply equivalent subjects  Search modes - Boolean/Phrase | 4,526 |
| S33 | (MH "Polymerase Chain Reaction") OR (MH "Primed In Situ Labeling") OR (MH "Random Amplified Polymorphic DNA Technique") OR (MH "Reverse Transcriptase Polymerase Chain Reaction") OR (MH "Blood Transfusion Reaction) | Expanders - Apply equivalent subjects  Search modes - Boolean/Phrase | 48,863 |
| S32 | (MH "Genotype") OR (MH "Phenotype") OR (MH "Genetic Markers") OR (MH "Polymorphism, Genetic") OR (MH "Alleles") OR (TX genetic N2 predisposition) OR (TX heterozygote* OR homozygote* OR phenotype* OR endophenotypes*) | Expanders - Apply equivalent subjects  Search modes - Boolean/Phrase | 104,816 |
| S31 | S29 OR S30 | Expanders - Apply equivalent subjects  Search modes - Boolean/Phrase | 104,745 |
| S30 | (MH "Blood Component Transfusion") OR (MH "Erythrocyte Transfusion") OR (MH "Exchange Transfusion, Whole Blood") OR (MH "Cytapheresis") OR (MH "Blood Transfusion") OR (MH "Blood Banks") | Expanders - Apply equivalent subjects  Search modes - Boolean/Phrase | 18,615 |
| S29 | (MH "Blood Grouping and Crossmatching") OR (MH "Hematologic Tests") OR (MH "Blood Groups") OR (MH "ABO Blood-Group System") OR (MH "Rh-Hr Blood-Group System") OR (MH "HLA Antigens") OR (TX blood N2 cell) OR (TX red N2 cell* N2 group*) OR (TX erythrocyte* N2 group*) OR (TX rbc N2 group*) OR (TX abo N2 group*) OR (TX "blood-group*") | Expanders - Apply equivalent subjects  Search modes - Boolean/Phrase | 91,204 |
| S28 | S26 OR S27 | Expanders - Apply equivalent subjects  Search modes - Boolean/Phrase | 16,782 |
| S27 | (TX thalassemi*) OR (TX sickle N2 anaemia*) OR (TX sickle N2 anemia*) OR (TX sickle N2 trait*) OR (TX sickle N2 cell*) OR (TX hemoglobinopath*) | Expanders - Apply equivalent subjects  Search modes - Boolean/Phrase | 16,775 |
| S26 | (MH "Hemoglobinopathies") OR (MH "Anemia, Sickle Cell") OR (MH "Acute Chest Syndrome") OR (MH "Sickle Cell Trait") OR (MH "Thalassemia") OR (MH "alpha-Thalassemia") OR (MH "beta-Thalassemia") OR (MH "delta-Thalassemia") | Expanders - Apply equivalent subjects  Search modes - Boolean/Phrase | 8,172 |
| S25 | S23 AND S24 | Expanders - Apply equivalent subjects  Search modes - Boolean/Phrase | 433 |
| S24 | EM 20150831*- | Expanders - Apply equivalent subjects  Search modes - Boolean/Phrase | 2,155,392 |
| S23 | S3 AND S11 AND S22 | Expanders - Apply equivalent subjects  Search modes - Boolean/Phrase | 1,388 |
| S22 | S12 OR S13 OR S14 OR S15 OR S16 OR S17 OR S18 OR S19 OR S20 OR S21 | Expanders - Apply equivalent subjects  Search modes - Boolean/Phrase | 262,520 |
| S21 | (TX donor* N2 select*) | Expanders - Apply equivalent subjects  Search modes - Boolean/Phrase | 1,301 |
| S20 | (MH "Blood Donors") OR (MH "Hematologic Tests") OR (MH "Blood Grouping and Crossmatching") | Expanders - Apply equivalent subjects  Search modes - Boolean/Phrase | 20,416 |
| S19 | (TX PCR OR RFLP OR ELISA OR bloodchip OR beadchip OR hea) OR (TX human N2 erythrocyt* N2 antigen*) OR (TX haemagglutinat* OR hemagglutinat* OR agglutinat*) OR (TX polymerase N2 chain N2 react*) OR (TX oligonucleotide N2 array*) OR (TX genotyp* OR phenotyp*) | Expanders - Apply equivalent subjects  Search modes - Boolean/Phrase | 210,501 |
| S18 | (MH "Enzyme-Linked Immunosorbent Assay") | Expanders - Apply equivalent subjects  Search modes - Boolean/Phrase | 22,193 |
| S17 | (MH "Microarray Analysis") OR (MH "Oligonucleotide Array Sequence Analysis") OR (MH "Protein Array Analysis") | Expanders - Apply equivalent subjects  Search modes - Boolean/Phrase | 4,526 |
| S16 | (MH "Blood Transfusion Reaction") | Expanders - Apply equivalent subjects  Search modes - Boolean/Phrase | 909 |
| S15 | (MH "Polymerase Chain Reaction") OR (MH "Primed In Situ Labeling") OR (MH "Random Amplified Polymorphic DNA Technique") OR (MH "Reverse Transcriptase Polymerase Chain Reaction") | Expanders - Apply equivalent subjects  Search modes - Boolean/Phrase | 47,963 |
| S14 | (TX genetic N2 predisposition) OR (TX heterozygote* OR homozygote* OR phenotype* OR endophenotypes*) | Expanders - Apply equivalent subjects  Search modes - Boolean/Phrase | 63,966 |
| S13 | (MH "Alleles") | Expanders - Apply equivalent subjects  Search modes - Boolean/Phrase | 9,050 |
| S12 | (MH "Genotype") OR (MH "Phenotype") OR (MH "Genetic Markers") OR (MH "Polymorphism, Genetic") OR (MH "Polymorphism, Single Nucleotide") | Expanders - Apply equivalent subjects  Search modes - Boolean/Phrase | 67,628 |
| S11 | S4 OR S5 OR S6 OR S7 OR S8 OR S9 OR S10 | Expanders - Apply equivalent subjects  Search modes - Boolean/Phrase | 104,745 |
| S10 | (MH "Blood Banks") | Expanders - Apply equivalent subjects  Search modes - Boolean/Phrase | 2,309 |
| S9 | (MH "Blood Component Transfusion") OR (MH "Erythrocyte Transfusion") OR (MH "Exchange Transfusion, Whole Blood") OR (MH "Cytapheresis") OR (MH "Blood Transfusion") | Expanders - Apply equivalent subjects  Search modes - Boolean/Phrase | 17,251 |
| S8 | (TX blood N2 cell) OR (TX red N2 cell* N2 group*) OR (TX erythrocyte* N2 group*) OR (TX rbc N2 group*) OR (TX abo N2 group*) OR (TX "blood-group*") | Expanders - Apply equivalent subjects  Search modes - Boolean/Phrase | 73,259 |
| S7 | (MH "HLA Antigens") | Expanders - Apply equivalent subjects  Search modes - Boolean/Phrase | 4,284 |
| S6 | (MH "Blood Groups") OR (MH "ABO Blood-Group System") OR (MH "Rh-Hr Blood-Group System") | Expanders - Apply equivalent subjects  Search modes - Boolean/Phrase | 2,492 |
| S5 | (MH "Hematologic Tests") | Expanders - Apply equivalent subjects  Search modes - Boolean/Phrase | 15,434 |
| S4 | (MH "Blood Grouping and Crossmatching") | Expanders - Apply equivalent subjects  Search modes - Boolean/Phrase | 843 |
| S3 | S1 OR S2 | Expanders - Apply equivalent subjects  Search modes - Boolean/Phrase | 16,782 |
| S2 | (TX thalassemi*) OR (TX sickle N2 anaemia*) OR (TX sickle N2 anemia*) OR (TX sickle N2 trait*) OR (TX sickle N2 cell*) OR (TX hemoglobinopath*) | Expanders - Apply equivalent subjects  Search modes - Boolean/Phrase | 16,775 |
| S1 | (MH "Hemoglobinopathies") OR (MH "Anemia, Sickle Cell") OR (MH "Acute Chest Syndrome") OR (MH "Sickle Cell Trait") OR (MH "Thalassemia") OR (MH "alpha-Thalassemia") OR (MH "beta-Thalassemia") OR (MH "delta-Thalassemia") | Expanders - Apply equivalent subjects  Search modes - Boolean/Phrase | 8,172 |

**PRISMA Initial Results:** total number of results before duplicates removed

| **Database [Platform]** Searches run August 2, 2023. *Results limited to years Mar 23, 2021 to current.* | **Results** |
| --- | --- |
| MEDLINE(R) ALL 1946 to August 01, 2023 [Ovid] | 111 |
| Embase Classic+Embase 1947 to 2023 August 01 [Ovid] | 1,017 |
| EBM Reviews - Cochrane Central Register of Controlled Trials June 2023 [Ovid] | 6 |
| CINAHL 1981 to current [EBSCOHost] | 255 |
| **TOTAL** | **1,389** |

**MEDLINE(R) ALL**1946 to August 01, 2023
Search Strategy:

| **#** | **Searches** | **Results** |
| --- | --- | --- |
| 1 | hemoglobinopathies/ or anemia, sickle cell/ or acute chest syndrome/ or hemoglobin sc disease/ or sickle cell trait/ or hemoglobin c disease/ or thalassemia/ or alpha-thalassemia/ or beta-thalassemia/ or delta-thalassemia/ | 48070 |
| 2 | (hemoglobinopath* or (sickle adj2 (cell or cells or trait* or anemia* or anaemia*)) or thalassemi*).mp. | 59869 |
| 3 | 1 or 2 | 60035 |
| 4 | blood group antigens/ or abo blood-group system/ or duffy blood-group system/ or i blood-group system/ or kell blood-group system/ or kidd blood-group system/ or lewis blood group antigens/ or lewis x antigen/ or sialyl lewis x antigen/ or ca-19-9 antigen/ or lutheran blood-group system/ or mnss blood-group system/ or p blood-group system/ or rh-hr blood-group system/ | 47955 |
| 5 | (((blood or (red adj2 cell) or erythrocyte* or rbc or abo) adj2 group*) or "blood-group*").mp. | 67208 |
| 6 | exp Blood Transfusion/ | 92377 |
| 7 | Blood Banks/ | 7494 |
| 8 | or/4-7 | 163132 |
| 9 | genotype/ or genetic predisposition to disease/ or heterozygote/ or homozygote/ or phenotype/ or endophenotypes/ or genetic markers/ or genetic pleiotropy/ | 663606 |
| 10 | polymerase chain reaction/ or amplified fragment length polymorphism analysis/ or multiplex polymerase chain reaction/ or primed in situ labeling/ or random amplified polymorphic dna technique/ or real-time polymerase chain reaction/ or reverse transcriptase polymerase chain reaction/ | 466277 |
| 11 | microarray analysis/ or oligonucleotide array sequence analysis/ | 79481 |
| 12 | enzyme-linked immunosorbent assay/ or enzyme-linked immunospot assay/ or enzyme multiplied immunoassay technique/ | 155550 |
| 13 | polymorphism, genetic/ or genomic structural variation/ or dna copy number variations/ or pharmacogenomic variants/ or polymorphism, restriction fragment length/ or polymorphism, single nucleotide/ or polymorphism, single-stranded conformational/ | 303422 |
| 14 | (PCR or RFLP or ELISA or bloodchip or beadchip or hea or (human adj2 erythrocyt* adj2 antigen*) or haemagglutinat* or hemagglutinat* or agglutinat* or (polymerase adj2 chain adj2 react*) or (oligonucleotide adj2 array*) or genotyp* or phenotyp*).ti,ab. | 1853812 |
| 15 | hematologic tests/ or "blood grouping and crossmatching"/ | 15043 |
| 16 | Donor Selection/ | 3799 |
| 17 | or/9-16 | 2485985 |
| 18 | 3 and 8 and 17 | 1046 |
| 19 | limit 18 to ed=20210323-20230801 | 100 |
| 20 | ("20210323" or "20210324" or "20210325" or "20210326" or "20210327" or "20210328" or "20210329" or "20210330" or "20210331" or "202104*" or "202105*" or "202106*" or "202107*" or "202108*" or "202109*" or "202110*" or "202111*" or "202112*" or "2022*" or "2023*").dt. | 3737595 |
| 21 | 18 and 20 | 77 |
| 22 | 19 or 21 | 111 |

**Embase Classic+Embase**1947 to 2023 August 01
Search Strategy:

| **#** | **Searches** | **Results** |
| --- | --- | --- |
| 1 | hemoglobinopathy/ or hemoglobin c disease/ | 11592 |
| 2 | sickle cell/ or acute chest syndrome/ | 7040 |
| 3 | sickle cell anemia/ or hemoglobin sc disease/ or hemoglobin sd disease/ or sickle cell beta thalassemia/ or sickle cell crisis/ or sickle cell trait/ | 49431 |
| 4 | thalassemia/ or alpha thalassemia/ or beta thalassemia/ or delta thalassemia/ or thalassemia intermedia/ or thalassemia major/ or thalassemia minor/ | 41725 |
| 5 | (hemoglobinopath* or (sickle adj2 (cell or cells or trait* or anemia* or anaemia*)) or thalassemi*).mp. | 97378 |
| 6 | or/1-5 | 97644 |
| 7 | blood group antigen/ or blood group a antigen/ or blood group abh antigen/ or blood group b antigen/ or blood group e antigen/ or blood group h antigen/ or blood group i antigen/ or blood group o antigen/ or rhesus antigen/ or rhesus d antigen/ or sialyl lewis x antigen/ | 22074 |
| 8 | blood group system/ or blood group abo system/ or blood group duffy system/ or blood group i system/ or blood group kell system/ or blood group kidd system/ or blood group lewis system/ or blood group lutheran system/ or blood group mnss system/ or blood group p system/ or blood group rhesus system/ or blood group xg system/ | 28769 |
| 9 | lymphocyte antigen/ or cd15 antigen/ | 10953 |
| 10 | CA 19-9 antigen/ | 21297 |
| 11 | (((blood or (red adj2 cell) or erythrocyte* or rbc or abo) adj2 group*) or "blood-group*").mp. | 97283 |
| 12 | blood bank/ | 16332 |
| 13 | blood transfusion/ or blood autotransfusion/ or exp blood component therapy/ or exchange blood transfusion/ | 231409 |
| 14 | or/7-13 | 358490 |
| 15 | genotype/ or haplotype/ | 514302 |
| 16 | genetic predisposition/ or genetic resistance/ or genetic susceptibility/ | 207738 |
| 17 | genetic parameters/ | 4256 |
| 18 | genetic code/ or exp codon/ | 125801 |
| 19 | genetic risk/ | 72285 |
| 20 | genetic similarity/ | 6764 |
| 21 | mutation rate/ | 23592 |
| 22 | population genetic parameters/ or familial incidence/ or gene frequency/ or genetic variability/ | 475966 |
| 23 | heterozygosity/ or heterozygote/ | 150087 |
| 24 | homozygote/ or homozygosity/ | 95112 |
| 25 | phenotype/ or phenotypic variation/ | 710347 |
| 26 | genetic marker/ or cleaved amplified polymorphic sequence/ or dna marker/ or marker chromosome/ or marker gene/ or microsatellite marker/ or random amplified microsatellite/ or sequence characterized amplified region/ | 120660 |
| 27 | random amplified polymorphic dna/ or random amplified polymorphic dna polymerase chain reaction/ | 8304 |
| 28 | sequence characterized amplified region/ | 275 |
| 29 | inheritance/ | 34197 |
| 30 | pleiotropy/ or antagonistic pleiotropy/ | 18528 |
| 31 | nucleic acid analysis/ or amplified fragment length polymorphism/ or branched dna signal amplification assay/ or chromatin immunoprecipitation/ or differential display/ or dna determination/ or dna extraction/ or dna gel blot analysis/ or dna isolation/ or dna screening/ or dot hybridization/ or heteroduplex analysis/ or high resolution melting analysis/ or inverse polymerase chain reaction/ or ligase chain reaction/ or loop mediated isothermal amplification/ or multiplex ligation dependent probe amplification/ or multiplex polymerase chain reaction/ or nick end labeling/ or northern blotting/ or nucleic acid amplification/ or nucleic acid sequence based amplification/ or polymerase chain reaction/ or primed in situ labeling/ or random amplified microsatellite/ or random amplified polymorphic dna/ or real time polymerase chain reaction/ or restriction fragment length polymorphism/ or reverse transcription loop mediated isothermal amplification/ or reverse transcription polymerase chain reaction/ or ribonuclease protection assay/ or rna analysis/ or rna gel blot analysis/ or sequence characterized amplified region/ or "serial analysis of gene expression"/ or single strand conformation polymorphism/ or slot blot hybridization/ or southern blotting/ or southwestern blotting/ or telomeric repeat amplification protocol/ | 1442348 |
| 32 | chromatin immunoprecipitation/ or chromatin immunoprecipitation polymerase chain reaction/ or chromatin immunoprecipitation sequencing/ | 48117 |
| 33 | polymerase chain reaction/ or inverse polymerase chain reaction/ | 494202 |
| 34 | multiplex ligation dependent probe amplification/ or methylation-specific multiplex ligation-dependent probe amplification/ | 8344 |
| 35 | multiplex polymerase chain reaction/ or multiplex real time polymerase chain reaction/ or multiplex reverse transcription polymerase chain reaction/ | 23797 |
| 36 | tunel assay/ | 28749 |
| 37 | random amplified polymorphic dna/ or random amplified polymorphic dna polymerase chain reaction/ | 8304 |
| 38 | real time polymerase chain reaction/ or allele specific real time polymerase chain reaction/ or fluorescence quantitative polymerase chain reaction/ or multiplex real time polymerase chain reaction/ or quantitative methylation specific polymerase chain reaction/ or real time reverse transcription polymerase chain reaction/ | 428497 |
| 39 | restriction fragment length polymorphism/ or polymerase chain reaction restriction fragment length polymorphism/ | 63541 |
| 40 | reverse transcription polymerase chain reaction/ or multiplex reverse transcription polymerase chain reaction/ | 310915 |
| 41 | microarray analysis/ or microchip analysis/ | 90648 |
| 42 | hemoglobin analysis/ or hemoglobin determination/ | 31314 |
| 43 | immunoassay/ | 84207 |
| 44 | DNA microarray/ | 67236 |
| 45 | protein analysis/ or protein microarray/ | 275945 |
| 46 | enzyme linked immunosorbent assay/ or ferritin elisa/ | 475637 |
| 47 | enzyme linked immunospot assay/ or enzyme multiplied immunoassay technique/ | 16788 |
| 48 | genetic polymorphism/ | 136716 |
| 49 | dna polymorphism/ or amplified fragment length polymorphism/ | 79875 |
| 50 | restriction fragment length polymorphism/ or polymerase chain reaction restriction fragment length polymorphism/ | 63541 |
| 51 | single nucleotide polymorphism/ | 240781 |
| 52 | protein polymorphism/ | 7257 |
| 53 | copy number variation/ | 35975 |
| 54 | (PCR or RFLP or ELISA or bloodchip or beadchip or hea or (human adj2 erythrocyt* adj2 antigen*) or haemagglutinat* or hemagglutinat* or agglutinat* or (polymerase adj2 chain adj2 react*) or (oligonucleotide adj2 array*) or genotyp* or phenotyp*).ti,ab. | 2532779 |
| 55 | blood examination/ or blood analysis/ or blood group typing/ | 113841 |
| 56 | antibody screening/ or dna screening/ or donor selection/ | 14851 |
| 57 | or/15-56 | 4585158 |
| 58 | 6 and 14 and 57 | 5208 |
| 59 | limit 58 to dc=20210323-20230801 | 1017 |

**EBM Reviews - Cochrane Central Register of Controlled Trials**June 2023
Search Strategy:

| **#** | **Searches** | **Results** |
| --- | --- | --- |
| 1 | hemoglobinopathies/ or anemia, sickle cell/ or acute chest syndrome/ or hemoglobin sc disease/ or sickle cell trait/ or hemoglobin c disease/ or thalassemia/ or alpha-thalassemia/ or beta-thalassemia/ or delta-thalassemia/ | 1390 |
| 2 | (hemoglobinopath* or (sickle adj2 (cell or cells or trait* or anemia* or anaemia*)) or thalassemi*).mp. | 3531 |
| 3 | hemoglobinopathy/ or hemoglobin c disease/ | 20 |
| 4 | sickle cell/ or acute chest syndrome/ | 76 |
| 5 | sickle cell anemia/ or hemoglobin sc disease/ or hemoglobin sd disease/ or sickle cell beta thalassemia/ or sickle cell crisis/ or sickle cell trait/ | 159 |
| 6 | thalassemia/ or alpha thalassemia/ or beta thalassemia/ or delta thalassemia/ or thalassemia intermedia/ or thalassemia major/ or thalassemia minor/ | 531 |
| 7 | (hemoglobinopath* or (sickle adj2 (cell or cells or trait* or anemia* or anaemia*)) or thalassemi*).mp. | 3531 |
| 8 | or/1-7 | 3534 |
| 9 | blood group antigens/ or abo blood-group system/ or duffy blood-group system/ or i blood-group system/ or kell blood-group system/ or kidd blood-group system/ or lewis blood-group system/ or antigens, cd15/ or ca-19-9 antigen/ or lutheran blood-group system/ or mnss blood-group system/ or p blood-group system/ or rh-hr blood-group system/ | 263 |
| 10 | blood group antigen/ or blood group a antigen/ or blood group abh antigen/ or blood group b antigen/ or blood group e antigen/ or blood group h antigen/ or blood group i antigen/ or blood group o antigen/ or rhesus antigen/ or rhesus d antigen/ or sialyl lewis x antigen/ | 32 |
| 11 | blood group system/ or blood group abo system/ or blood group duffy system/ or blood group i system/ or blood group kell system/ or blood group kidd system/ or blood group lewis system/ or blood group lutheran system/ or blood group mnss system/ or blood group p system/ or blood group rhesus system/ or blood group xg system/ | 16 |
| 12 | lymphocyte antigen/ or cd15 antigen/ | 10 |
| 13 | CA 19-9 antigen/ | 55 |
| 14 | (((blood or (red adj2 cell) or erythrocyte* or rbc or abo) adj2 group*) or "blood-group*").mp. | 5966 |
| 15 | exp blood transfusion/ | 4792 |
| 16 | Blood Banks/ | 50 |
| 17 | blood bank/ | 21 |
| 18 | blood transfusion/ or blood autotransfusion/ or exp blood component therapy/ or exchange blood transfusion/ | 2790 |
| 19 | or/9-18 | 10530 |
| 20 | genotype/ or genetic predisposition to disease/ or heterozygote/ or homozygote/ or phenotype/ or endophenotypes/ or genetic markers/ or genetic pleiotropy/ | 8888 |
| 21 | polymerase chain reaction/ or amplified fragment length polymorphism analysis/ or multiplex polymerase chain reaction/ or primed in situ labeling/ or random amplified polymorphic dna technique/ or real-time polymerase chain reaction/ or reverse transcriptase polymerase chain reaction/ | 3281 |
| 22 | microarray analysis/ or oligonucleotide array sequence analysis/ | 367 |
| 23 | enzyme-linked immunosorbent assay/ or enzyme-linked immunospot assay/ or enzyme multiplied immunoassay technique/ | 4311 |
| 24 | polymorphism, genetic/ or genomic structural variation/ or dna copy number variations/ or pharmacogenomic variants/ or polymorphism, restriction fragment length/ or polymorphism, single nucleotide/ or polymorphism, single-stranded conformational/ | 4238 |
| 25 | (PCR or RFLP or ELISA or bloodchip or beadchip or hea or (human adj2 erythrocyt* adj2 antigen*) or haemagglutinat* or hemagglutinat* or agglutinat* or (polymerase adj2 chain adj2 react*) or (oligonucleotide adj2 array*) or genotyp* or phenotyp*).ti,ab. | 49151 |
| 26 | hematologic tests/ or "blood grouping and crossmatching"/ | 269 |
| 27 | Donor Selection/ | 48 |
| 28 | genotype/ or haplotype/ | 5472 |
| 29 | genetic predisposition/ or genetic resistance/ or genetic susceptibility/ | 439 |
| 30 | genetic parameters/ | 3 |
| 31 | genetic code/ or exp codon/ | 144 |
| 32 | genetic risk/ | 82 |
| 33 | genetic similarity/ | 2 |
| 34 | mutation rate/ | 44 |
| 35 | population genetic parameters/ or familial incidence/ or gene frequency/ or genetic variability/ | 1100 |
| 36 | heterozygosity/ or heterozygote/ | 540 |
| 37 | homozygote/ or homozygosity/ | 416 |
| 38 | phenotype/ or phenotypic variation/ | 2111 |
| 39 | genetic marker/ or cleaved amplified polymorphic sequence/ or dna marker/ or marker chromosome/ or marker gene/ or microsatellite marker/ or random amplified microsatellite/ or sequence characterized amplified region/ | 612 |
| 40 | random amplified polymorphic dna/ or random amplified polymorphic dna polymerase chain reaction/ | 1 |
| 41 | sequence characterized amplified region/ | 0 |
| 42 | inheritance/ | 11 |
| 43 | pleiotropy/ or antagonistic pleiotropy/ | 69 |
| 44 | nucleic acid analysis/ or amplified fragment length polymorphism/ or branched dna signal amplification assay/ or chromatin immunoprecipitation/ or differential display/ or dna determination/ or dna extraction/ or dna gel blot analysis/ or dna isolation/ or dna screening/ or dot hybridization/ or heteroduplex analysis/ or high resolution melting analysis/ or inverse polymerase chain reaction/ or ligase chain reaction/ or loop mediated isothermal amplification/ or multiplex ligation dependent probe amplification/ or multiplex polymerase chain reaction/ or nick end labeling/ or northern blotting/ or nucleic acid amplification/ or nucleic acid sequence based amplification/ or polymerase chain reaction/ or primed in situ labeling/ or random amplified microsatellite/ or random amplified polymorphic dna/ or real time polymerase chain reaction/ or restriction fragment length polymorphism/ or reverse transcription loop mediated isothermal amplification/ or reverse transcription polymerase chain reaction/ or ribonuclease protection assay/ or rna analysis/ or rna gel blot analysis/ or sequence characterized amplified region/ or "serial analysis of gene expression"/ or single strand conformation polymorphism/ or slot blot hybridization/ or southern blotting/ or southwestern blotting/ or telomeric repeat amplification protocol/ | 3000 |
| 45 | chromatin immunoprecipitation/ or chromatin immunoprecipitation polymerase chain reaction/ or chromatin immunoprecipitation sequencing/ | 5 |
| 46 | polymerase chain reaction/ or inverse polymerase chain reaction/ | 1934 |
| 47 | multiplex ligation dependent probe amplification/ or methylation-specific multiplex ligation-dependent probe amplification/ | 4 |
| 48 | multiplex polymerase chain reaction/ or multiplex real time polymerase chain reaction/ or multiplex reverse transcription polymerase chain reaction/ | 59 |
| 49 | tunel assay/ | 49 |
| 50 | random amplified polymorphic dna/ or random amplified polymorphic dna polymerase chain reaction/ | 1 |
| 51 | real time polymerase chain reaction/ or allele specific real time polymerase chain reaction/ or fluorescence quantitative polymerase chain reaction/ or multiplex real time polymerase chain reaction/ or quantitative methylation specific polymerase chain reaction/ or real time reverse transcription polymerase chain reaction/ | 680 |
| 52 | restriction fragment length polymorphism/ or polymerase chain reaction restriction fragment length polymorphism/ | 33 |
| 53 | reverse transcription polymerase chain reaction/ or multiplex reverse transcription polymerase chain reaction/ | 157 |
| 54 | microarray analysis/ or microchip analysis/ | 101 |
| 55 | hemoglobin analysis/ or hemoglobin determination/ | 115 |
| 56 | immunoassay/ | 486 |
| 57 | DNA microarray/ | 43 |
| 58 | protein analysis/ or protein microarray/ | 81 |
| 59 | enzyme linked immunosorbent assay/ or ferritin elisa/ | 4168 |
| 60 | enzyme linked immunospot assay/ or enzyme multiplied immunoassay technique/ | 173 |
| 61 | genetic polymorphism/ | 176 |
| 62 | dna polymorphism/ or amplified fragment length polymorphism/ | 170 |
| 63 | restriction fragment length polymorphism/ or polymerase chain reaction restriction fragment length polymorphism/ | 33 |
| 64 | single nucleotide polymorphism/ | 278 |
| 65 | protein polymorphism/ | 2 |
| 66 | copy number variation/ | 19 |
| 67 | blood examination/ or blood analysis/ or blood group typing/ | 284 |
| 68 | antibody screening/ or dna screening/ or donor selection/ | 59 |
| 69 | or/20-68 | 57841 |
| 70 | 8 and 19 and 69 | 35 |
| 71 | limit 70 to yr="2021 -Current" | 6 |

**CINAHL**

| **#** | **Query** | **Limiters/Expanders** | **Results** |
| --- | --- | --- | --- |
| S1 | (MH "Hemoglobinopathies") OR (MH "Anemia, Sickle Cell") OR (MH "Acute Chest Syndrome") OR (MH "Sickle Cell Trait") OR (MH "Thalassemia") OR (MH "alpha-Thalassemia") OR (MH "beta-Thalassemia") OR (MH "delta-Thalassemia") | Expanders - Apply equivalent subjects Search modes - Boolean/Phrase | 9,099 |
| S2 | (TX thalassemi*) OR (TX sickle N2 anaemia*) OR (TX sickle N2 anemia*) OR (TX sickle N2 trait*) OR (TX sickle N2 cell*) OR (TX hemoglobinopath*) | Expanders - Apply equivalent subjects Search modes - Boolean/Phrase | 22,691 |
| S3 | S1 OR S2 | Expanders - Apply equivalent subjects Search modes - Boolean/Phrase | 22,699 |
| S4 | (MH "Blood Grouping and Crossmatching") | Expanders - Apply equivalent subjects Search modes - Boolean/Phrase | 907 |
| S5 | (MH "Hematologic Tests") | Expanders - Apply equivalent subjects Search modes - Boolean/Phrase | 16,226 |
| S6 | (MH "Blood Groups") OR (MH "ABO Blood-Group System") OR (MH "Rh-Hr Blood-Group System") | Expanders - Apply equivalent subjects Search modes - Boolean/Phrase | 2,775 |
| S7 | (MH "HLA Antigens") | Expanders - Apply equivalent subjects Search modes - Boolean/Phrase | 4,659 |
| S8 | (TX blood N2 cell) OR (TX red N2 cell* N2 group*) OR (TX erythrocyte* N2 group*) OR (TX rbc N2 group*) OR (TX abo N2 group*) OR (TX "blood-group*") | Expanders - Apply equivalent subjects Search modes - Boolean/Phrase | 102,097 |
| S9 | (MH "Blood Component Transfusion") OR (MH "Erythrocyte Transfusion") OR (MH "Exchange Transfusion, Whole Blood") OR (MH "Cytapheresis") OR (MH "Blood Transfusion") | Expanders - Apply equivalent subjects Search modes - Boolean/Phrase | 18,676 |
| S10 | (MH "Blood Banks") | Expanders - Apply equivalent subjects Search modes - Boolean/Phrase | 2,363 |
| S11 | S4 OR S5 OR S6 OR S7 OR S8 OR S9 OR S10 | Expanders - Apply equivalent subjects Search modes - Boolean/Phrase | 135,149 |
| S12 | (MH "Genotype") OR (MH "Phenotype") OR (MH "Genetic Markers") OR (MH "Polymorphism, Genetic") OR (MH "Polymorphism, Single Nucleotide") | Expanders - Apply equivalent subjects Search modes - Boolean/Phrase | 76,104 |
| S13 | (MH "Alleles") | Expanders - Apply equivalent subjects Search modes - Boolean/Phrase | 10,030 |
| S14 | (TX genetic N2 predisposition) OR (TX heterozygote* OR homozygote* OR phenotype* OR endophenotypes*) | Expanders - Apply equivalent subjects Search modes - Boolean/Phrase | 80,848 |
| S15 | (MH "Polymerase Chain Reaction") OR (MH "Primed In Situ Labeling") OR (MH "Random Amplified Polymorphic DNA Technique") OR (MH "Reverse Transcriptase Polymerase Chain Reaction") | Expanders - Apply equivalent subjects Search modes - Boolean/Phrase | 54,576 |
| S16 | (MH "Blood Transfusion Reaction") | Expanders - Apply equivalent subjects Search modes - Boolean/Phrase | 1,022 |
| S17 | (MH "Microarray Analysis") OR (MH "Oligonucleotide Array Sequence Analysis") OR (MH "Protein Array Analysis") | Expanders - Apply equivalent subjects Search modes - Boolean/Phrase | 5,019 |
| S18 | (MH "Enzyme-Linked Immunosorbent Assay") | Expanders - Apply equivalent subjects Search modes - Boolean/Phrase | 24,967 |
| S19 | (TX PCR OR RFLP OR ELISA OR bloodchip OR beadchip OR hea) OR (TX human N2 erythrocyt* N2 antigen*) OR (TX haemagglutinat* OR hemagglutinat* OR agglutinat*) OR (TX polymerase N2 chain N2 react*) OR (TX oligonucleotide N2 array*) OR (TX genotyp* OR phenotyp*) | Expanders - Apply equivalent subjects Search modes - Boolean/Phrase | 267,816 |
| S20 | (MH "Blood Donors") OR (MH "Hematologic Tests") OR (MH "Blood Grouping and Crossmatching") | Expanders - Apply equivalent subjects Search modes - Boolean/Phrase | 21,526 |
| S21 | (TX donor* N2 select*) | Expanders - Apply equivalent subjects Search modes - Boolean/Phrase | 1,929 |
| S22 | S12 OR S13 OR S14 OR S15 OR S16 OR S17 OR S18 OR S19 OR S20 OR S21 | Expanders - Apply equivalent subjects Search modes - Boolean/Phrase | 326,749 |
| S23 | S3 AND S11 AND S22 | Expanders - Apply equivalent subjects Search modes - Boolean/Phrase | 1,944 |
| S24 | EM 20160831*- | Expanders - Apply equivalent subjects Search modes - Boolean/Phrase | 2,630,909 |
| S25 | S23 AND S24 | Expanders - Apply equivalent subjects Search modes - Boolean/Phrase | 708 |
| S26 | EM 20210323*- | Expanders - Apply equivalent subjects Search modes - Boolean/Phrase | 845,719 |
| S27 | S25 AND S26 | Expanders - Apply equivalent subjects Search modes - Boolean/Phrase | 255 |

Bottom of Form

# Appendix E- GRADE definitions

| Certainty of evidence | Definition | Strength of recommendation |
| --- | --- | --- |
| High(A) | We are very confident that the true effect lies close to that of the estimate of the effect | Strong^[[1]](#footnote-2)^ |
| Moderate(B) | We are moderately confident in the effect estimate: The true effect is likely to be close to the estimate of the effect but there is a possibility that it is substantially different. | Fair |
| Low (C) | Our confidence in the effect estimate is limited: The true effect may be substantially different from the estimate of the effect. | Weak/conditional |
| Very Low (D) | We have very little confidence in the effect estimate: The true effect is likely to be substantially different from the estimate of effect. | Weak/conditional |
| **Source: Adapted from GRADE Handbook** | | |

# Appendix F- Disclosures table

| **Panel member** | **Conflict of interest** |
| --- | --- |
| Julia Wolf | No financial or intellectual conflicts of interest to declare. |
| Isabelle Blais-Normandin | No financial or intellectual conflicts of interest to declare. |
| Aarti Bathla | Financial disclosure, employee of Canadian Blood Services. |
| Homa Keshavarz | Financial disclosure, employee of Canadian Blood Services. |
| Stella T. Chou | Financial disclosures:  Royalties - Up to Date chapter; Honoraria - Grifols , Ortho diagnostics, American Society of Hematology Patent on red cell reagents; Chair - ASH transfusion guidelines for Sickle Cell Disease (SCD); Member - NIH SCD Advisory committee; Member - AABB Clinical Transfusion Medicine Committee; Member - OASH DHHS RBC Alloantibody Registry Exchange Committee; Received research grants – NIH; Pending research grants – NIH.  Intellectual disclosures:  SCD and transfusion – ASH.  Family member (Spouse) position/work - Jefferson Hospital System. |
| Arwa Al Riyami | Intellectual disclosures:  Chair, Clinical Transfusion Working Party – ISBT; Member, Platelet guidelines development group – ICTMG. |
| Cassandra Josephson | Financial disclosures:  Paid consultancies: Octapharma, Immucor; Other: Medtronics. Elected positions NHLBI – Co-chair REDS-IV-P Program, AABB Transfusion journal – Associate Editor. Intellectual disclosures – Research grant – NHLBI; Pending research grant – NHLBI. |
| Edwin Massey | Intellectual disclosures: Trompeter S., et al. Position paper on International Collaboration for Transfusion Medicine (ICTM) Guideline ‘Red blood cell specifications for patients with hemoglobinopathies: a systematic review and guideline. BJHaem 2020; 189(3):424-427; Transfusion Task Force – British Society of Haematology. |
| Heather Hume | Financial disclosures:  NIH Observational Monitoring Board for Bloodsafe studies, Président - Comité consultatif national en medécine transfusionnelle - advisory committee to Quebec Ministry of Health.  Other disclosures:  non-remunerated:  1) Sickle Pan-African Research Consortium (SPARCO) Uganda. Strengthening Capacity for Clinical Care, Research and Training in Sickle Cell Disease SCRT Project (Co-investigator). Sponsor: USA NIH.  2)  Randomized Trial to Evaluate the Mirasol Whole Blood Pathogen Reduction Technology System to Reduce Malaria and Emerging Transfusion Transmitted Infections U.S. Department of Defense Medical Research Program. |
| Jacob Pendergast | Financial disclosures:  Paid consultancies – GBT, Novartis, CSL Behring, Novo Nordisk; Officer - Sickle Cell Awareness Group of Ontario; Officer - Sickle Cell Disease Association of Canada; Officer - Thalassemia Foundation of Canada. |
| Greg Denomme | Financial disclosures:  Advisory board - Hema-Quebec Medical and Scientific Advisory Board; Honoraria - Abbott Transfusion Health Institute; Member - ISBT RBC terminology working group; Committee Chair - AABB - Molecular Testing Standards committee; Member – AABB – Standards Program Committee.  Intellectual disclosures:  Opinion or commentary -   1. Clausen FB., et al. Recommendation for validation and quality assurance of non-invasive prenatal testing for foetal blood groups. Vox Sang 2022; 117:157-165. 2. Denomme GA. Phage display reloaded: on the development of reliable monoclonal antibodies for potential Rh immune globulin production. Blood Transfus 2021; 19:3-4. 3. Flegel WA., et al. It is time to phase out "serologic weak D phenotype" and resolve D types with RHD genotyping including weak D type 4. Transfusion 2020; 60:855-859 4. Denomme GA, Anani WQ. ABO titers: harmonization and identifying clinically relevant ABO antibodies. Transfusion 2020; 60:441-443. |
| Rada M. Grubovic Rastvorceva | Intellectual disclosure: President of Macedonian Society for Transfusion Medicine. |
| Sara Trompeter | Intellectual disclosures: Research Grant – NHSBT; Opinion or Commentary - Trompeter S., et al. Position paper on International Collaboration for Transfusion Medicine (ICTM) Guideline ‘Red blood cell specifications for patients with hemoglobinopathies: a systematic review and guideline. BJHaem 2020; 189(3):424-427; Membership in a previous guideline panel – BSH – Apheresis, BSH – Sick cell and transfusion. |
| Simon Stanworth | Financial disclosures: Received research grant (Co-applicant on a national NIHR funded study on AI approaches to matching of blood for sickle cell disease). |

1. Recommendation 2 in the guideline manuscript is a strong recommendation, with low certainty of evidence and is an exception to the criteria defined above. The rationale behind this is provided under the evidence summary and rationale of recommendation 2. [↑](#footnote-ref-2)
